# Supplementary material for: Pleiotropic genetic architecture and novel loci for C-reactive protein levels
Source: Nat Commun. 2022 Nov 14;13:6939. doi: 10.1038/s41467-022-34688-6 (PMC9663411; doi:10.1038/s41467-022-34688-6)
Supplement: Supplementary file 1 — Supplementary Information [file 41467_2022_34688_MOESM1_ESM.pdf]

Supplementary Figures

Supplementary Figure 1 Venn diagrams of A) 295 C-reactive protein (CRP) independent SNPs which are also genome-wide significantly associated with any of the other examined traits (high density lipoprotein levels, HDL; low density lipoprotein levels, LDL; triglyceride levels, TG; body mass index BMI; cigarettes per day, CPD) in multi-trait MTAG and B) the same focusing on the novel CRP SNPs only

Supplementary Figure 2 Circular dendrogram presenting the novel C-reactive protein (CRP) loci Starting from the center, the layers represent the chromosome, rsID, MTAG analysis (1 = multi-trait, 2 = bivariate on CRP-lipids, 3 = bivariate on CRP-BMI), and the mapped genes, respectively. The loci from multi-trait MTAG are colored with blue and from bivariate with red

Supplementary Figure 3 Regional plots of the 41 novel C-reactive protein (CRP) loci comparing the results from the MTAG (blue) with the respective from the univariate UKB-CHARGE meta-analysis (yellow). The P-values come from two-sided statistical tests.

Supplementary Figure 4 Venn diagram of C-Reactive Protein (CRP) associated genes which are also associated with any of High-Density Lipoprotein (HDL), Low-Density Lipoprotein (LDL), Triglycerides (TG), Body Mass Index (BMI), and Cigarettes per day (CPD)

Supplementary Figure 5 Tissue expression analysis of a) 30 general and b) 54 more specific tissue types obtained from Genotype-Tissue Expression version 8 (GTEx v8) for C-Reactive Protein (CRP), Body Mass Index (BMI), High-Density Lipoprotein (HDL), Low-Density Lipoprotein (LDL), Triglycerides (TG), and Cigarettes per day (CPD) using MAGMA gene-property test. The P-values come from two-sided statistical tests. Horizontal line represents the Bonferroni threshold (a: $P=1.7\times10^{-3}$; b: $P=9.3\times10^{-4}$) and Bonferroni significant tissues are colored with red

Supplementary Figure 6 Venn diagram of the 41 candidate causal variants which colocalize between C-Reactive Protein levels (CRP) and any of the other examined traits: High-Density Lipoprotein (HDL), Low-Density Lipoprotein (LDL), Triglycerides (TG), Body Mass Index (BMI), and Cigarettes per day (CPD)

Supplementary Figure 7 Regional plots of the 41 loci found to colocalize between C-Reactive Protein levels (CRP) and any of the other examined traits: High-Density Lipoprotein (HDL), Low-Density Lipoprotein (LDL), Triglycerides (TG), Body Mass Index (BMI), and Cigarettes per day (CPD). The P-values come from two-sided statistical tests.

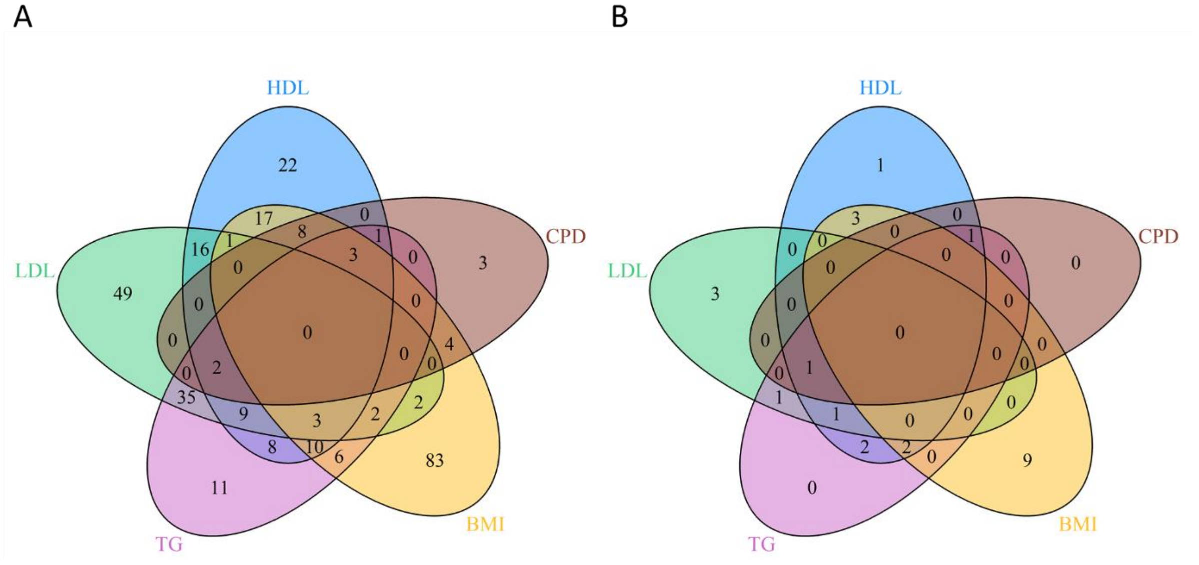

Supplementary Figure 1 *Venn diagrams of A) 295 C-reactive protein (CRP) independent SNPs which are also genome-wide significantly associated with any of the other examined traits (high density lipoprotein levels, HDL; low density lipoprotein levels, LDL; triglyceride levels, TG; body mass index BMI; cigarettes per day, CPD) in multi-trait MTAG and B) the same focusing on the novel CRP SNPs only*

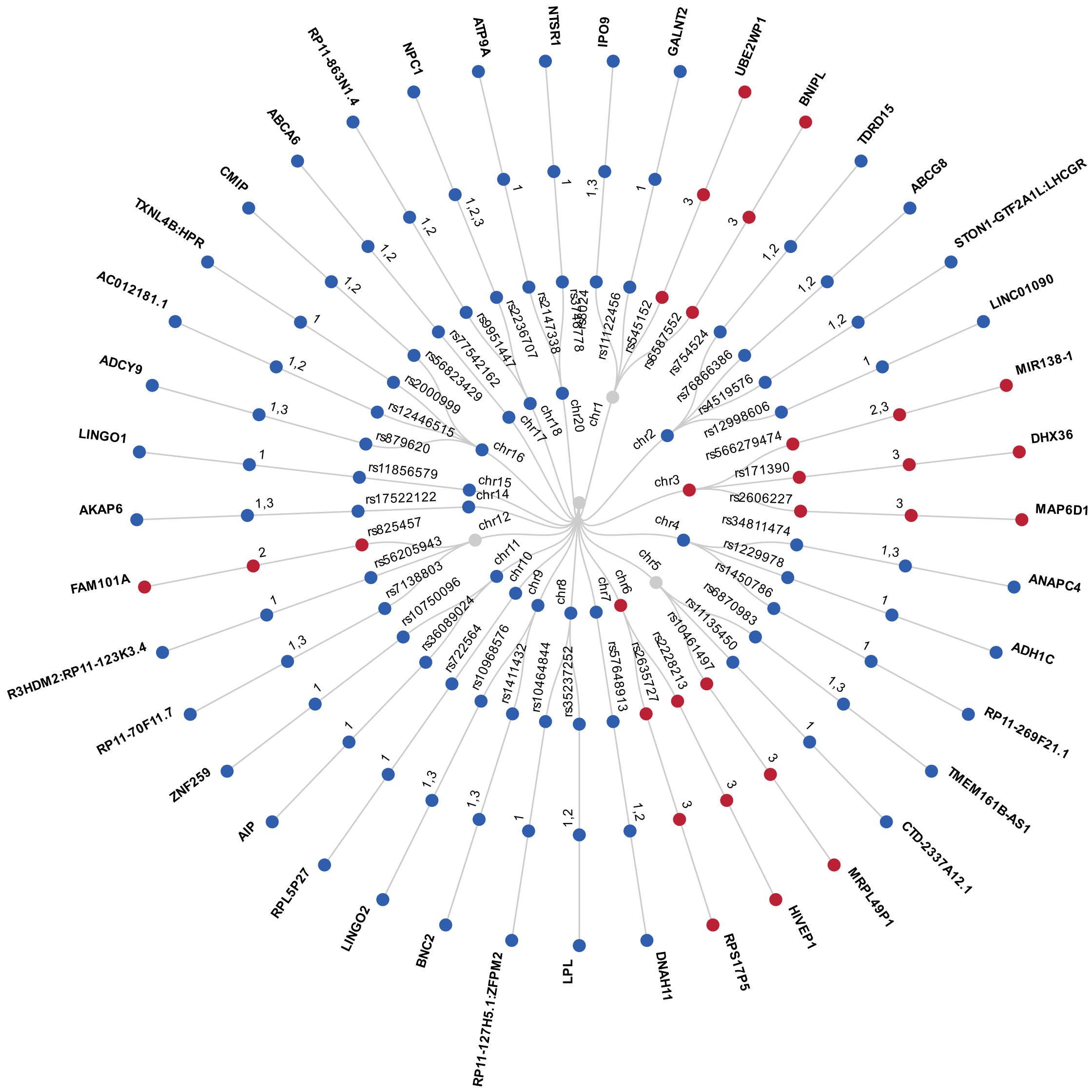

Supplementary Figure 2 *Circular dendrogram presenting the novel C-reactive protein (CRP) loci Starting from the center, the layers represent the chromosome, rsID, MTAG analysis (1 = multi-trait, 2 = bivariate on CRP-lipids, 3 = bivariate on CRP-BMI), and the mapped genes, respectively. The loci from multi-trait MTAG are colored with blue and from bivariate with red*

BMI = Body mass index; CPD = Cigarettes per day; CRP = C-reactive protein; HDL = High-Density lipoprotein; LDL = Low-Density lipoprotein; TG = Triglycerides

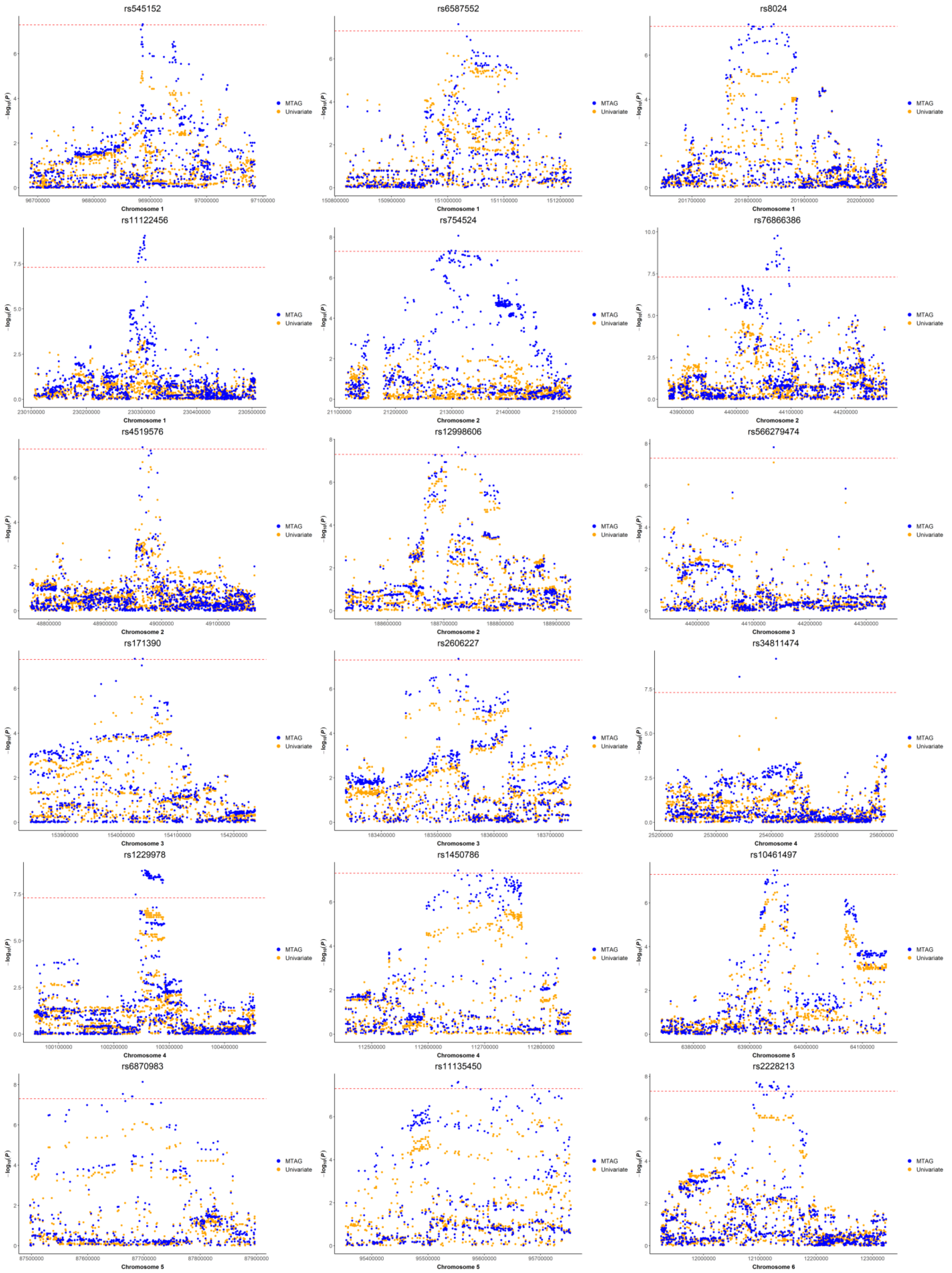
rs545152
Chromosome 1
rs6587552
Chromosome 1
rs8024
Chromosome 1
rs11122456
Chromosome 1
rs754524
Chromosome 2
rs76866386
Chromosome 2
rs4519576
Chromosome 2
rs12998606
Chromosome 2
rs566279474
Chromosome 3
rs171390
Chromosome 3
rs2606227
Chromosome 3
rs34811474
Chromosome 4
rs1229978
Chromosome 4
rs1450786
Chromosome 4
rs10461497
Chromosome 5
rs6870983
Chromosome 5
rs11135450
Chromosome 5
rs2228213
Chromosome 6
$-\log_{10}(P)$
MTAG
Univariate

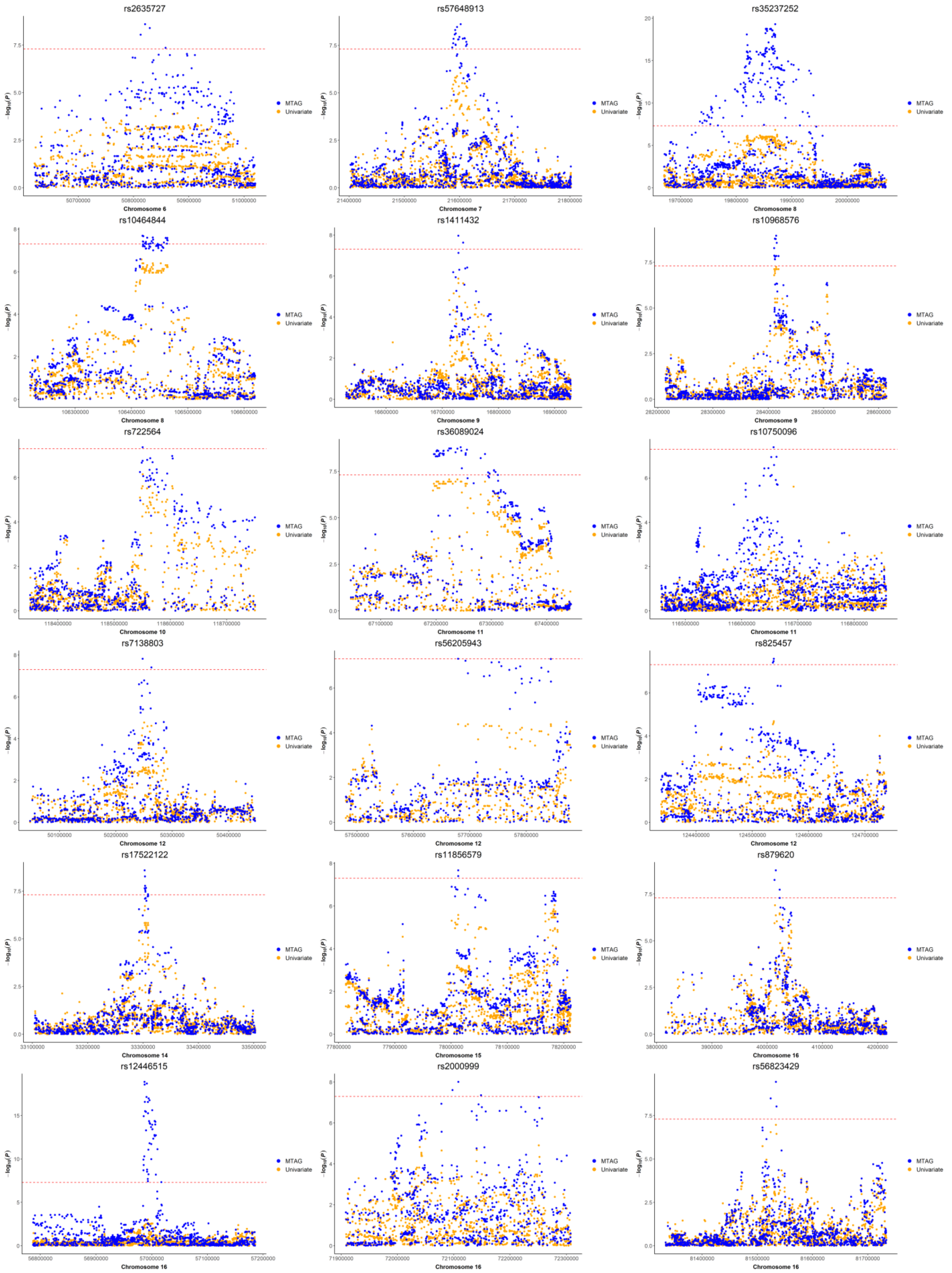
rs2635727
Chromosome 6
rs57648913
Chromosome 7
rs35237252
Chromosome 8
rs10464844
Chromosome 8
rs1411432
Chromosome 9
rs10968576
Chromosome 9
rs722564
Chromosome 10
rs36089024
Chromosome 11
rs10750096
Chromosome 11
rs7138803
Chromosome 12
rs56205943
Chromosome 12
rs825457
Chromosome 12
rs17522122
Chromosome 14
rs11856579
Chromosome 15
rs879620
Chromosome 16
rs12446515
Chromosome 16
rs2000999
Chromosome 16
rs56823429
Chromosome 16
MTAG
Univariate
−log10(P)

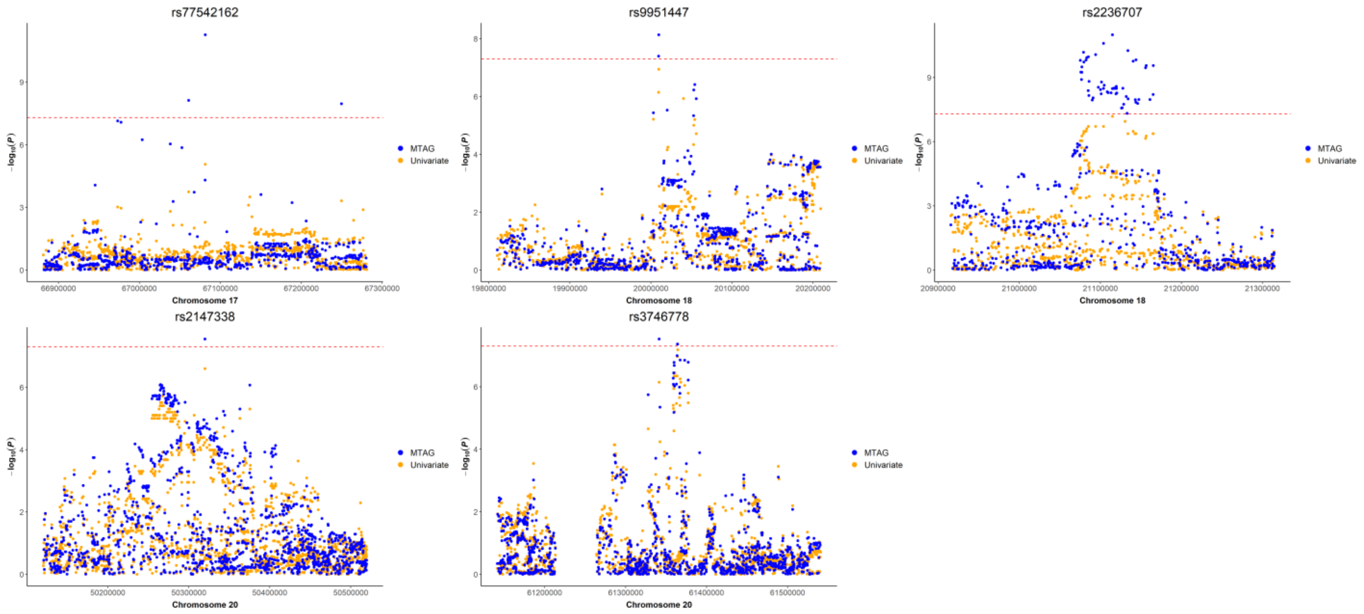

Supplementary Figure 3 *Regional plots of the 41 novel C-reactive protein (CRP) loci comparing the results from the MTAG (blue) with the respective from the univariate UKB-CHARGE meta-analysis (yellow). The P-values come from two-sided statistical tests.*

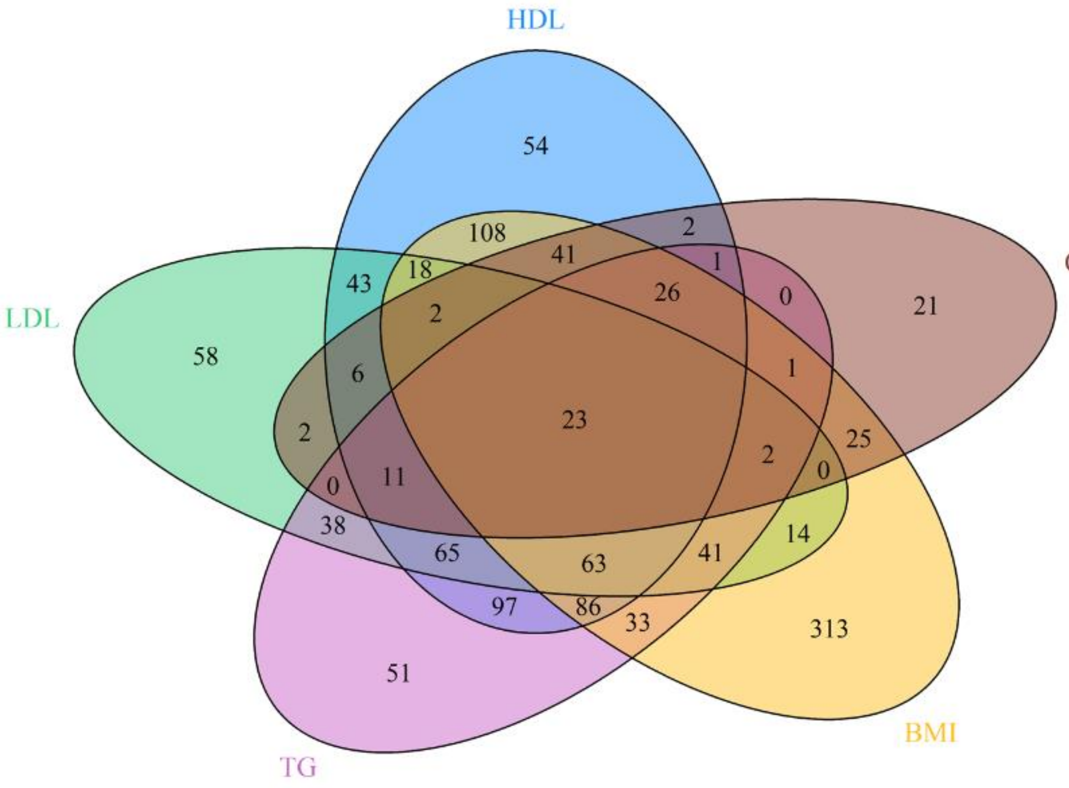

Supplementary Figure 4 *Venn diagram of C-Reactive Protein (CRP) associated genes which are also associated with any of High-Density Lipoprotein (HDL), Low-Density Lipoprotein (LDL), Triglycerides (TG), Body Mass Index (BMI), and Cigarettes per day (CPD)*

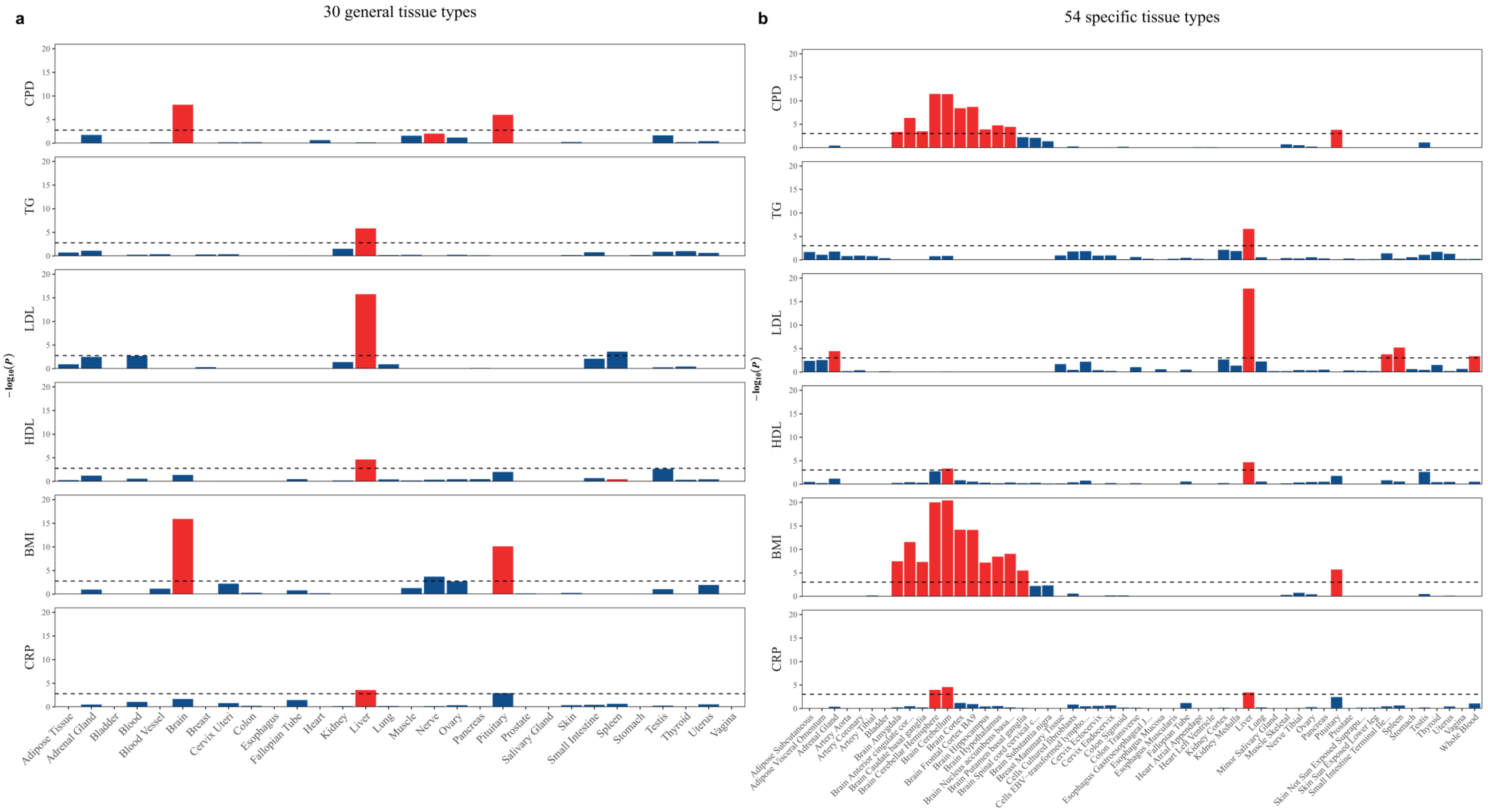

Supplementary Figure 5 *Tissue expression analysis of a) 30 general and b) 54 more specific tissue types obtained from Genotype-Tissue Expression version 8 (GTEx v8) for C-Reactive Protein (CRP), Body Mass Index (BMI), High-Density Lipoprotein (HDL), Low-Density Lipoprotein (LDL), Triglycerides (TG), and Cigarettes per day (CPD) using MAGMA gene-property test. The P-values come from two-sided statistical tests. Horizontal line represents the Bonferroni threshold (a:* $P=1.7\times10^{-3}$*; b:* $P=9.3\times10^{-4}$*) and Bonferroni significant tissues are colored with red*

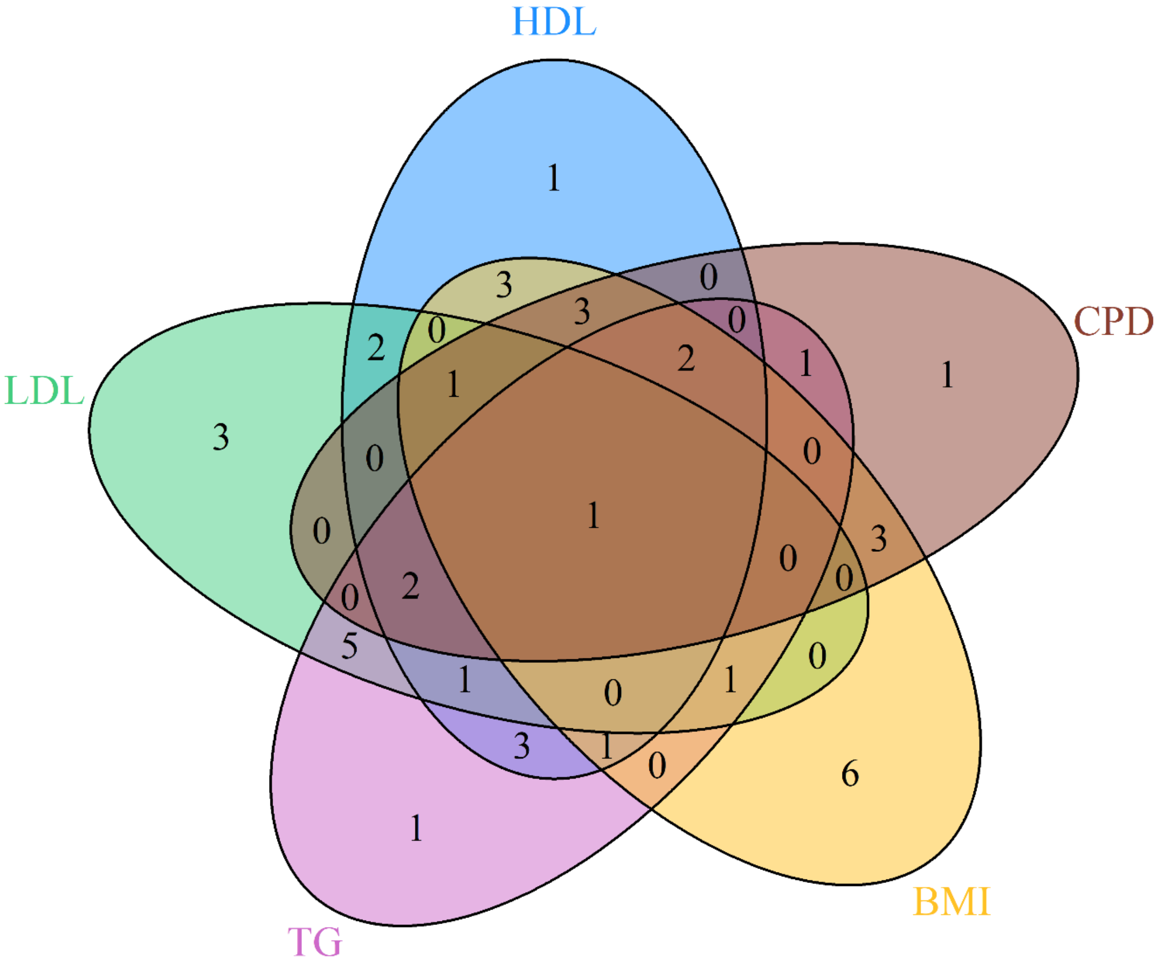

Supplementary Figure 6 *Venn diagram of the 41 candidate causal variants which colocalize between C-Reactive Protein levels (CRP) and any of the other examined traits: High-Density Lipoprotein (HDL), Low-Density Lipoprotein (LDL), Triglycerides (TG), Body Mass Index (BMI), and Cigarettes per day (CPD)*

rs75460349

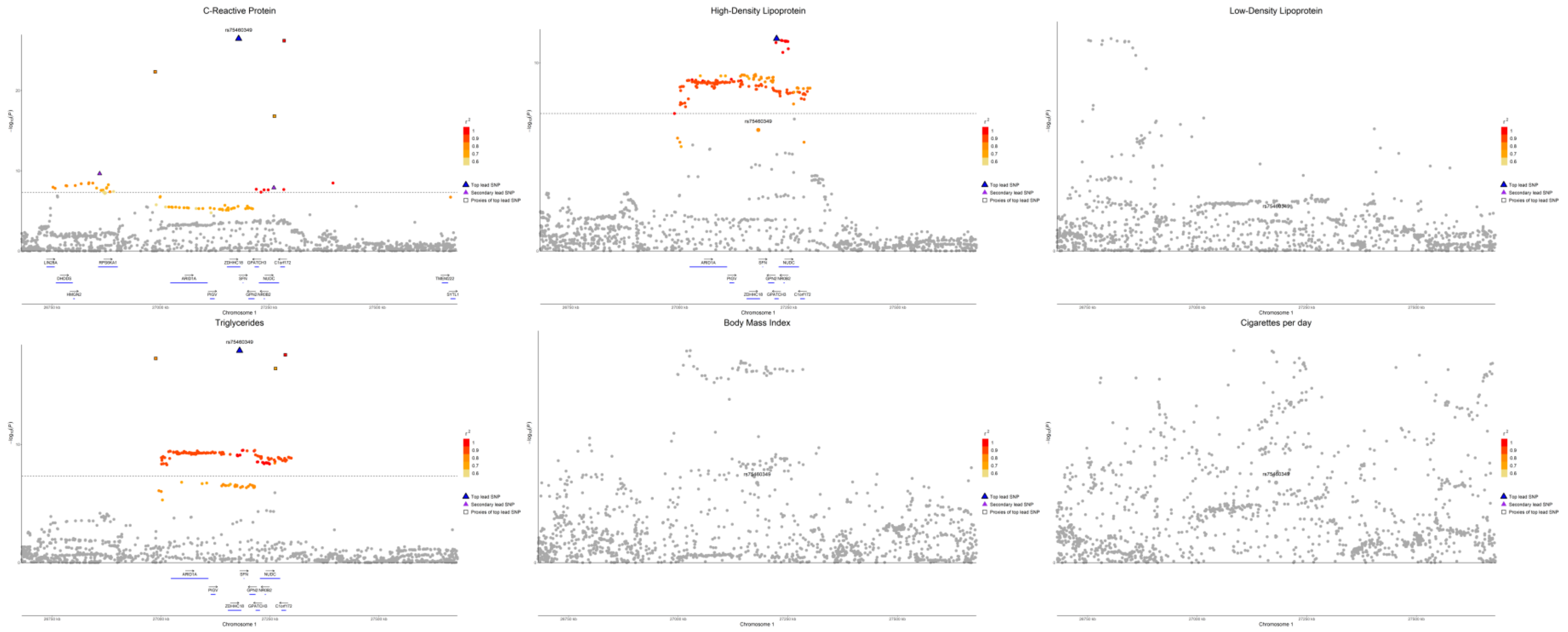

rs61812598

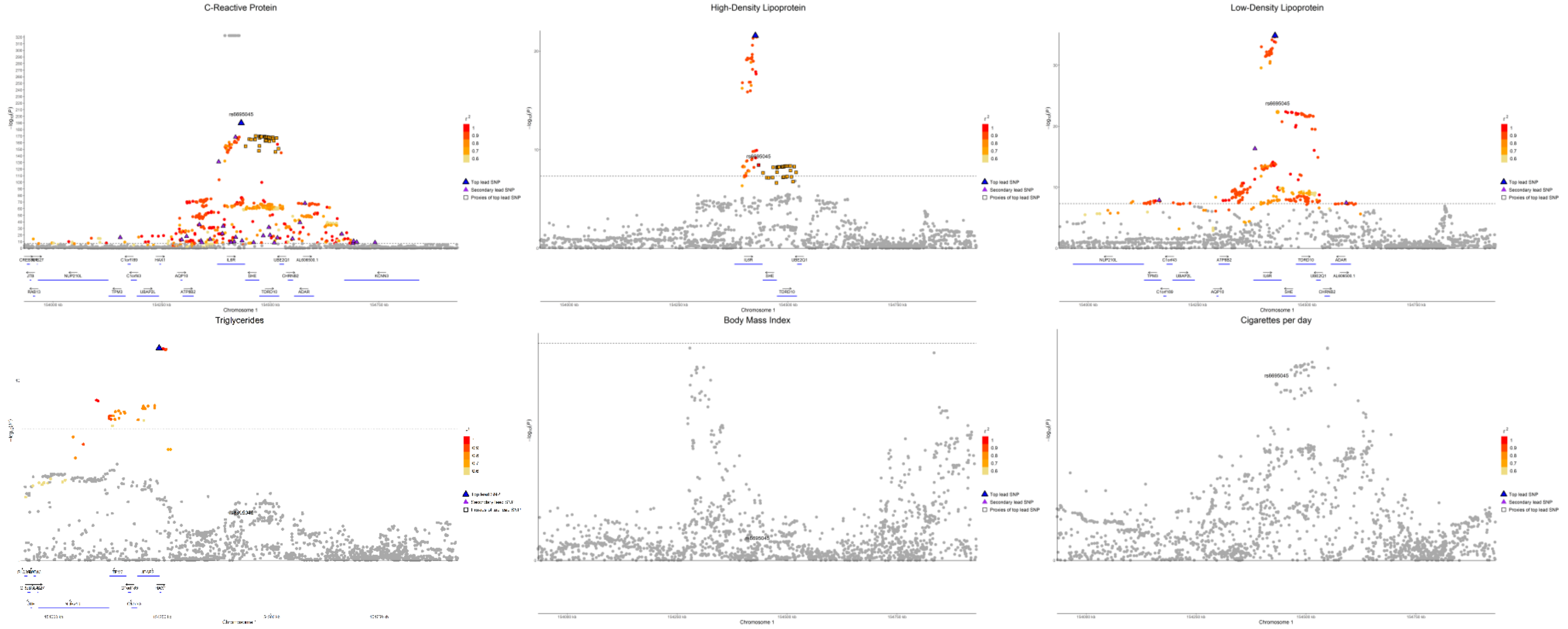

rs2211320

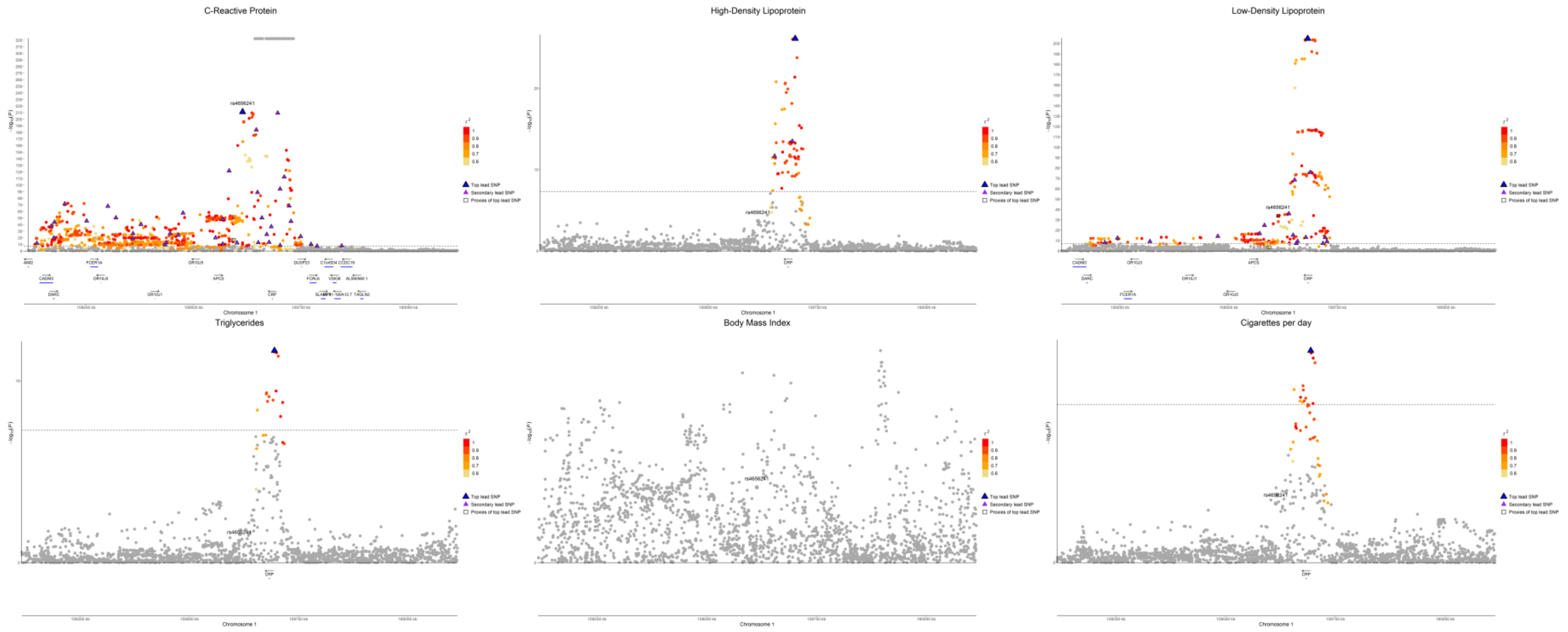

rs4658403

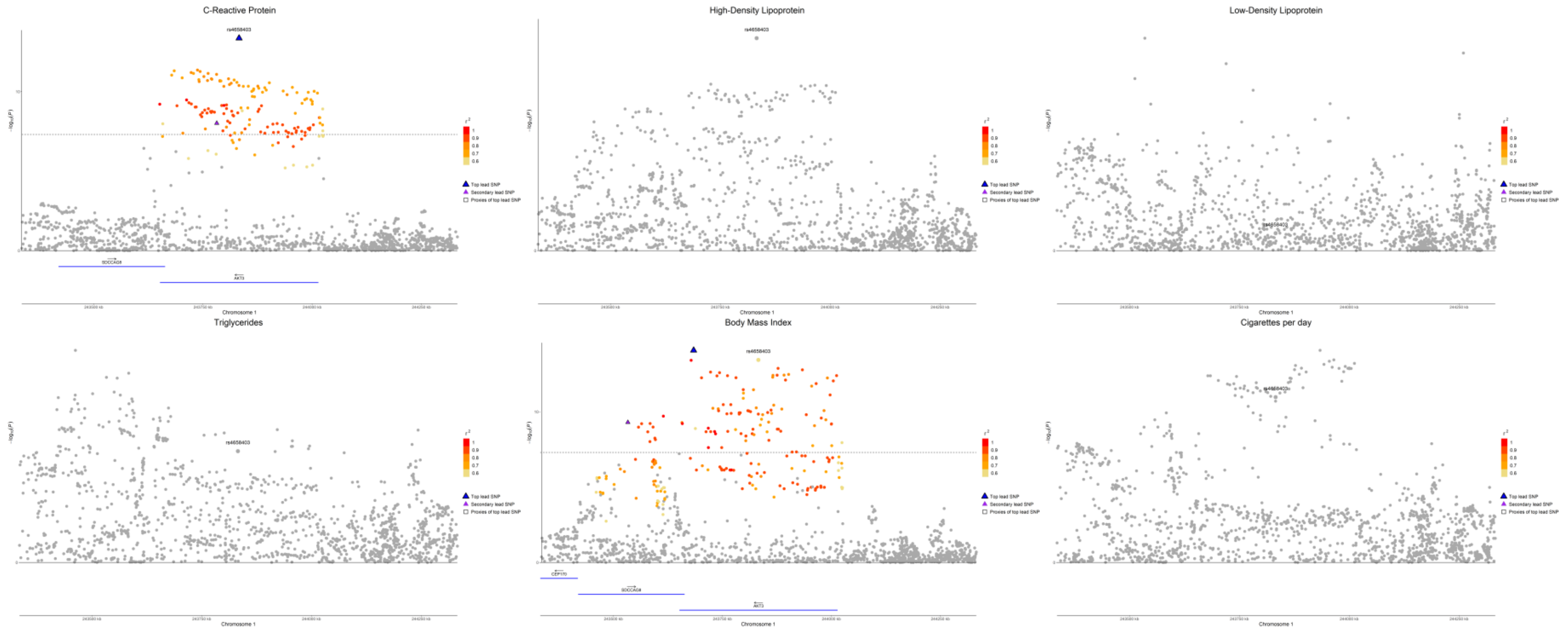

rs1260326

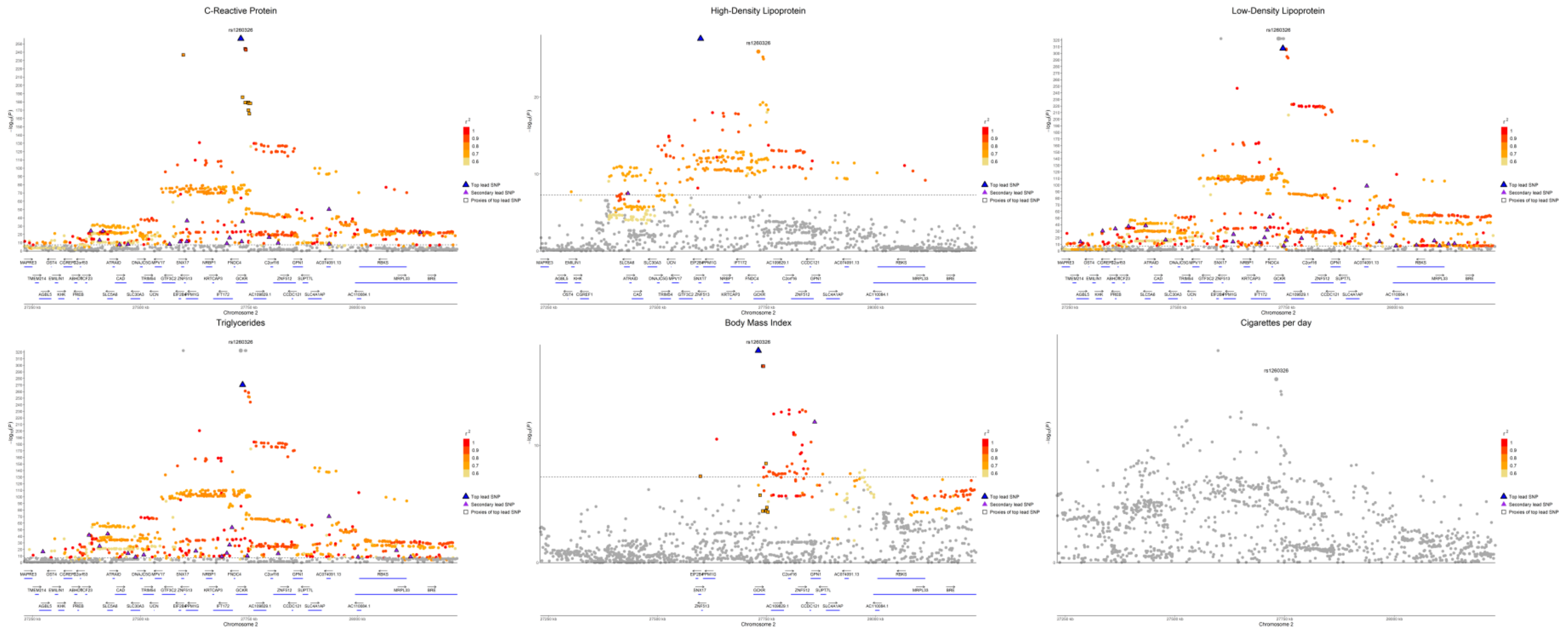

rs17326656

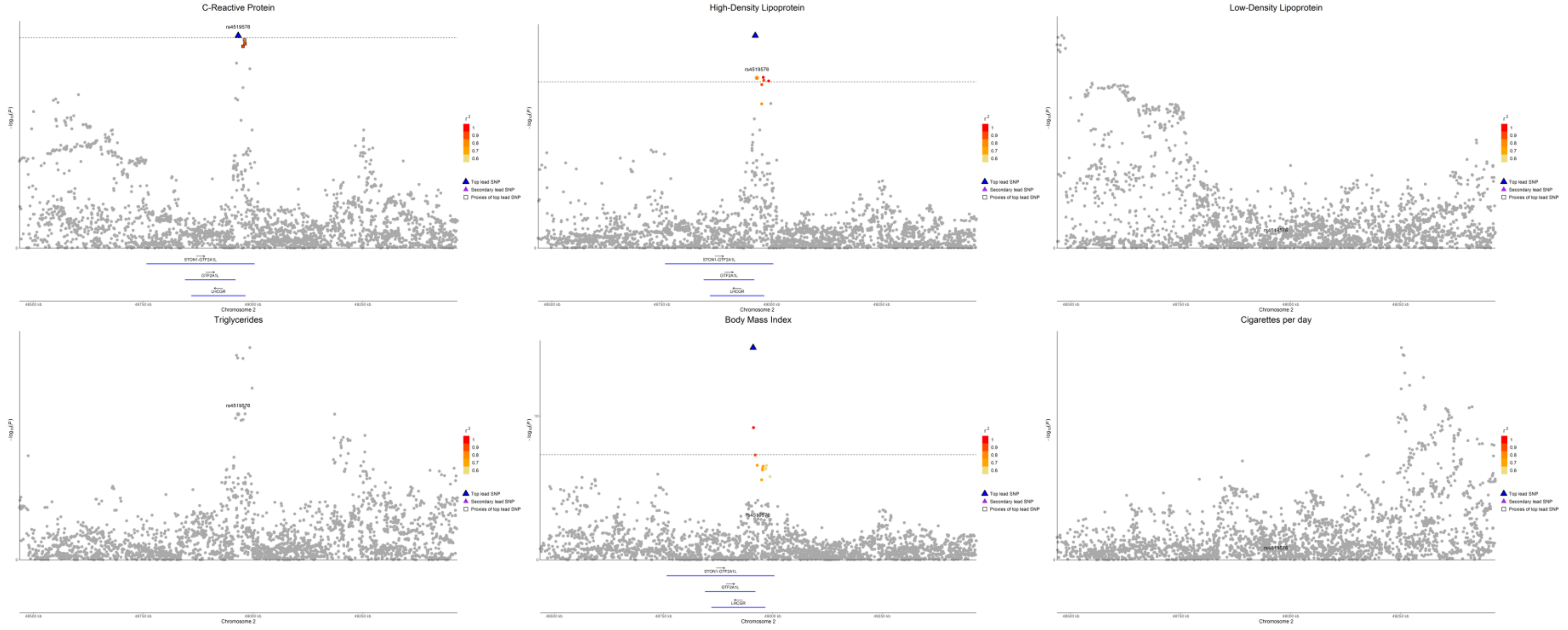

rs2161037

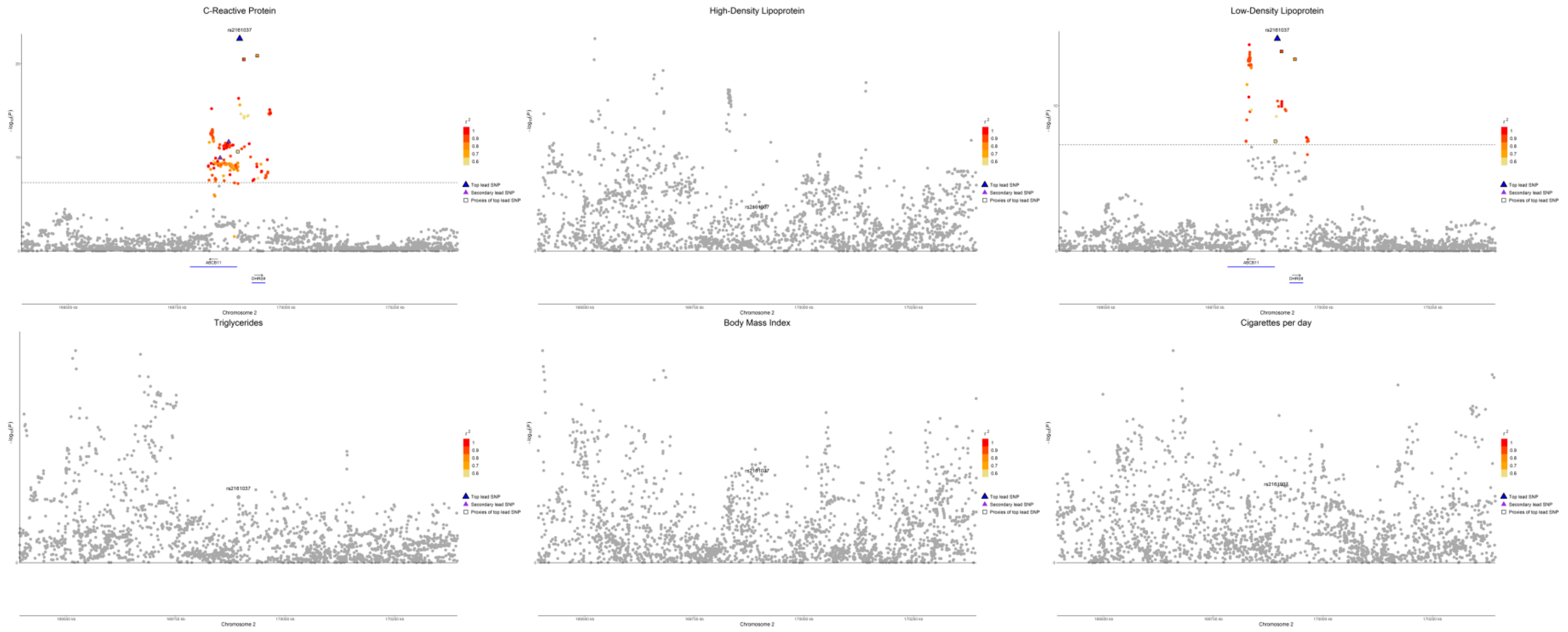

rs6792725

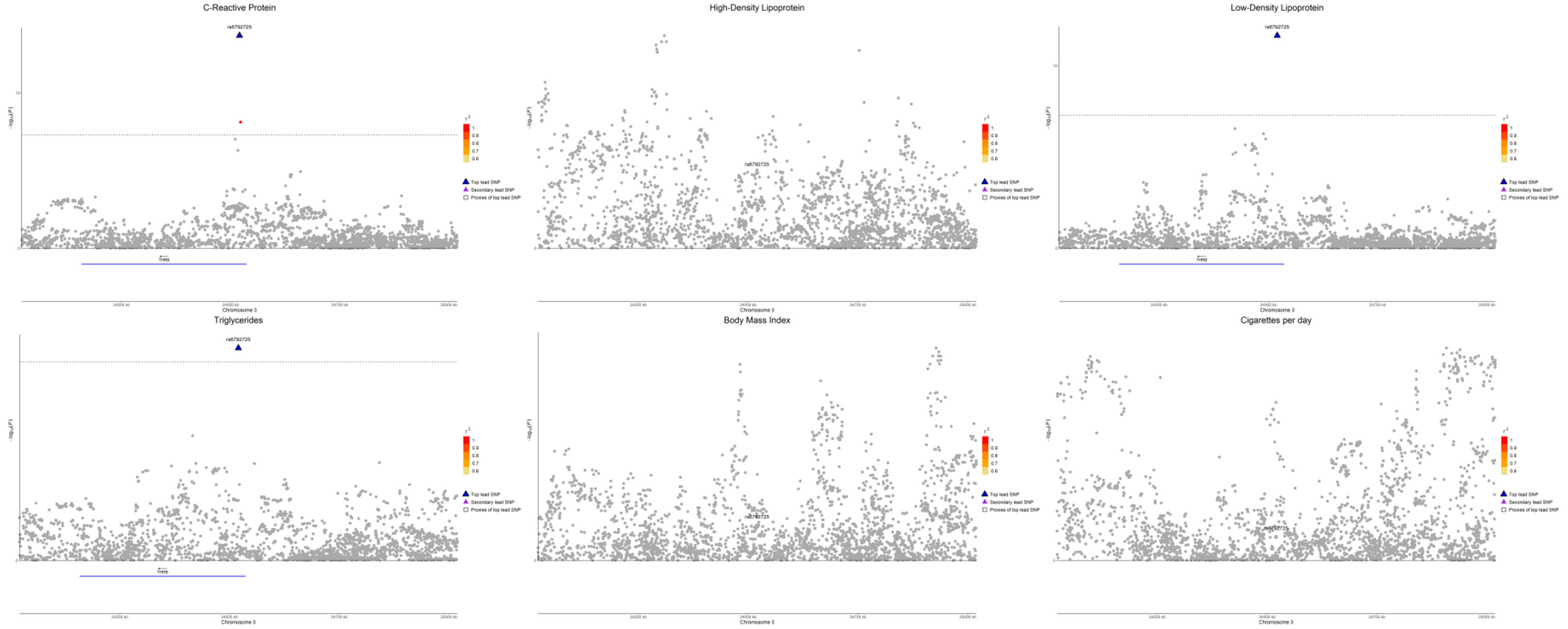

rs171390

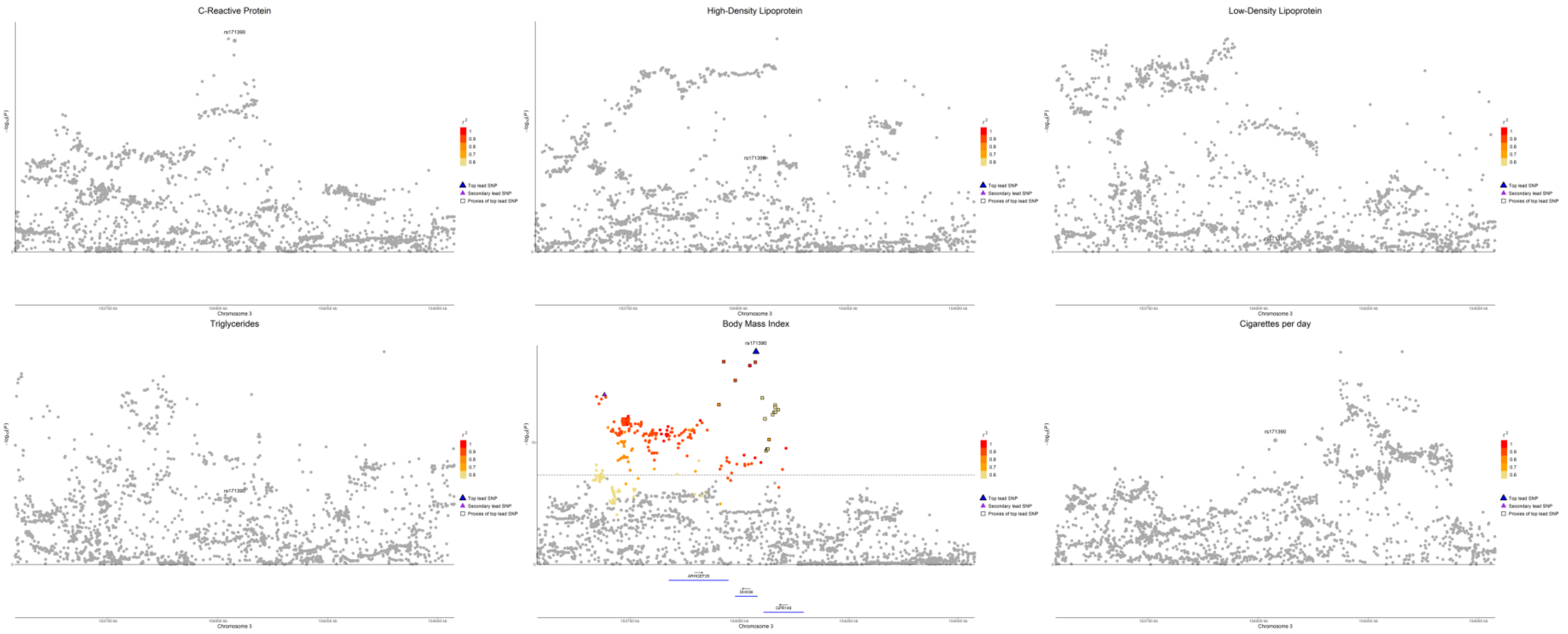

rs247975

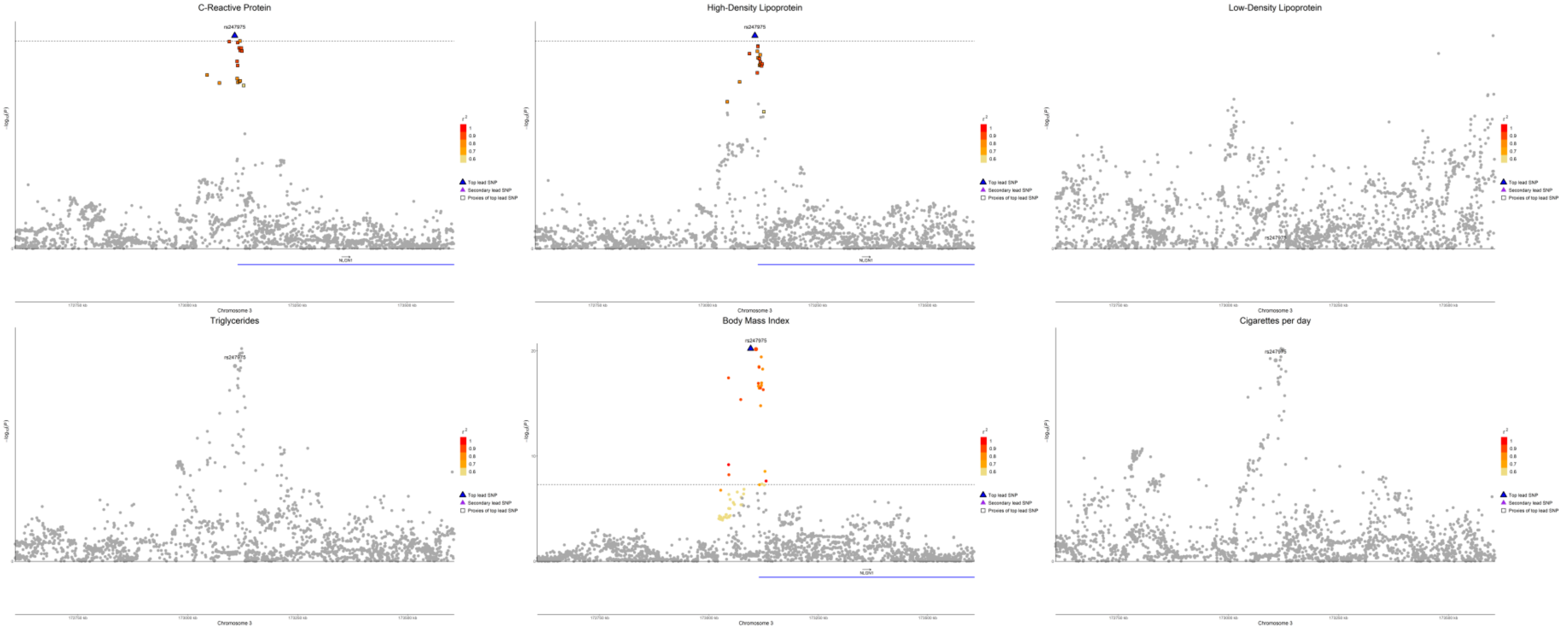

rs34811474

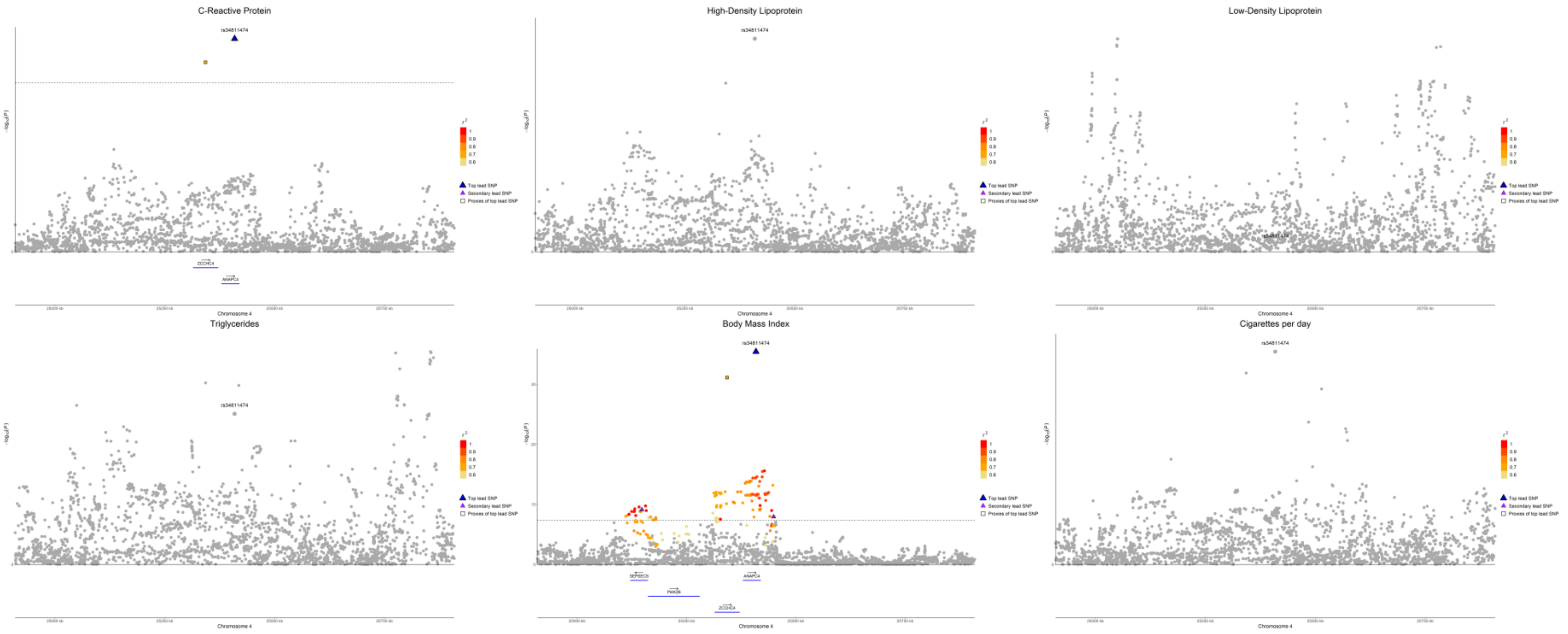

rs10938397

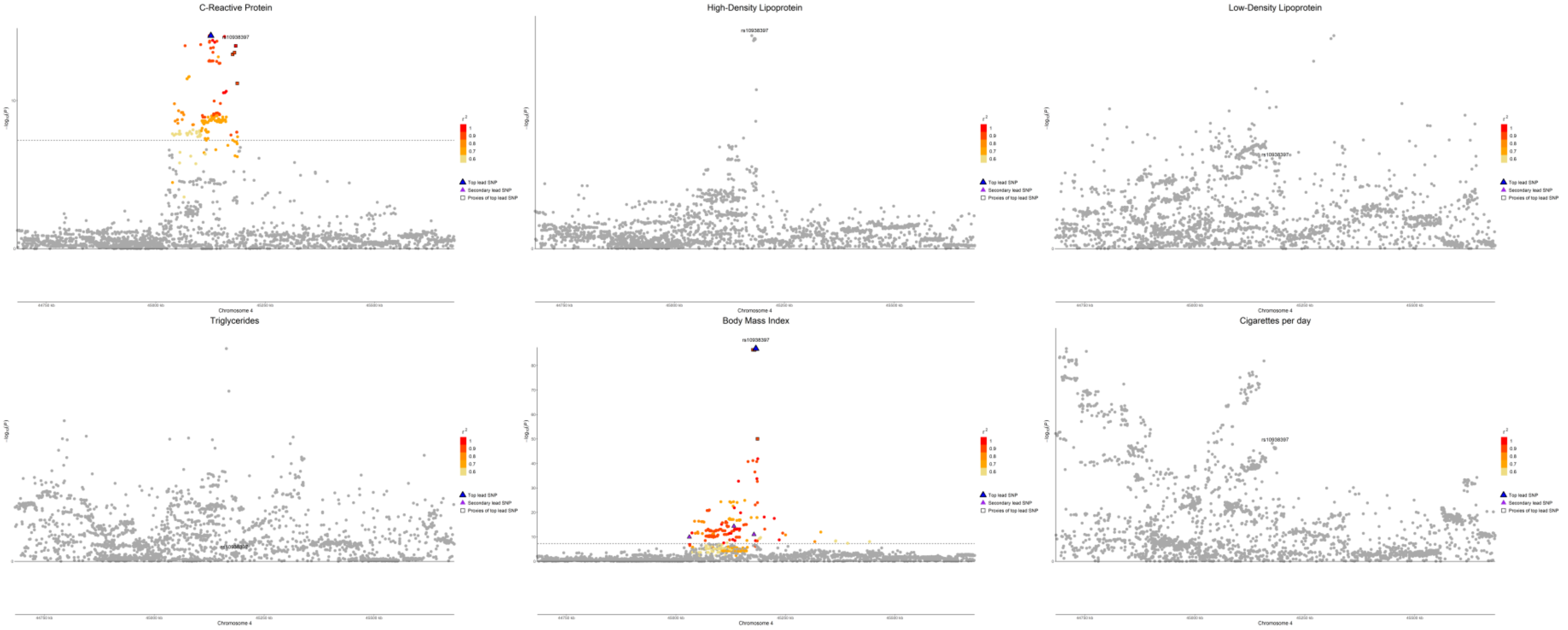

rs6870983

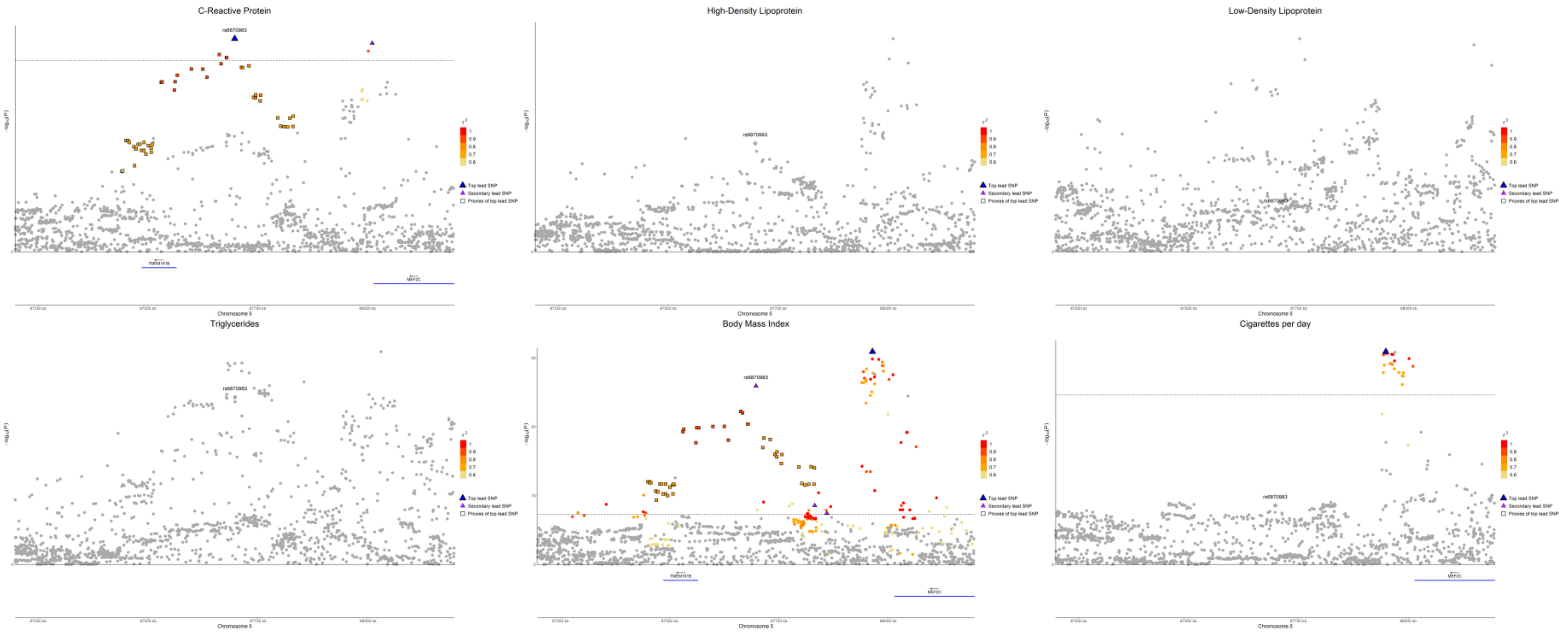

rs2228213

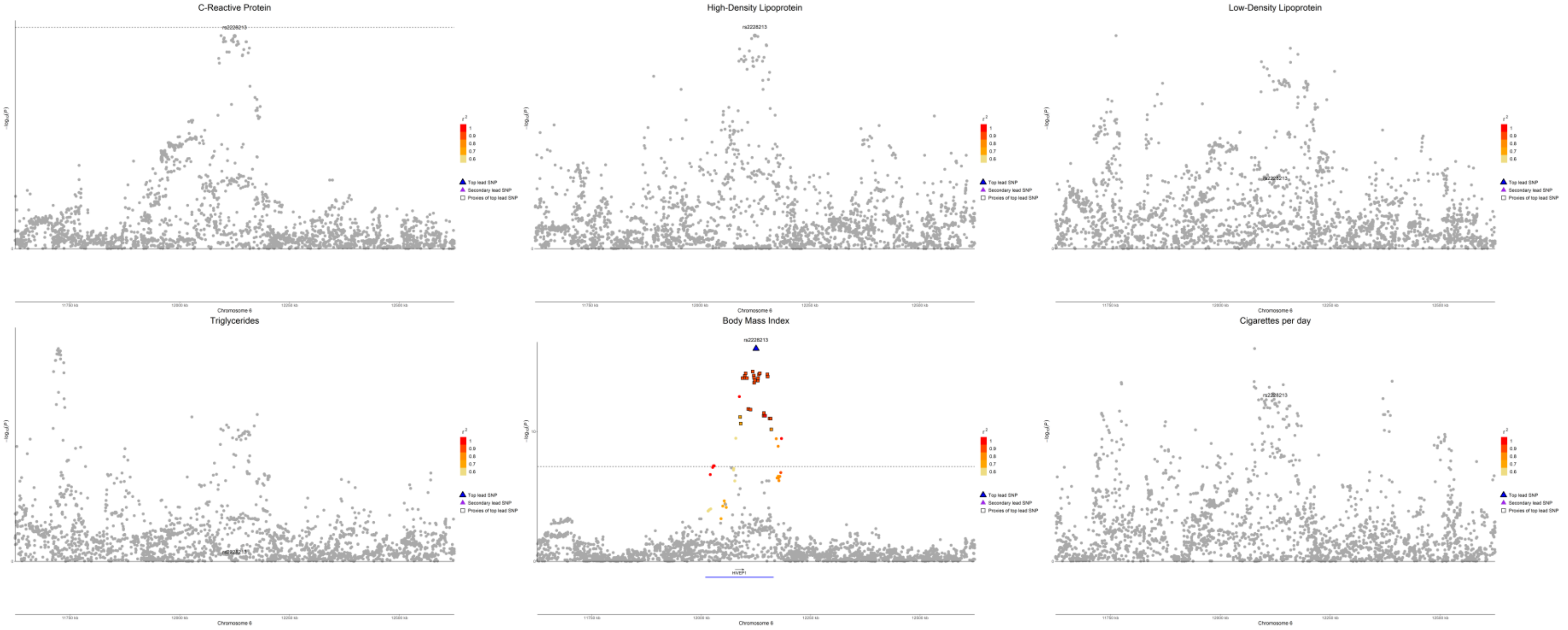

rs5017416

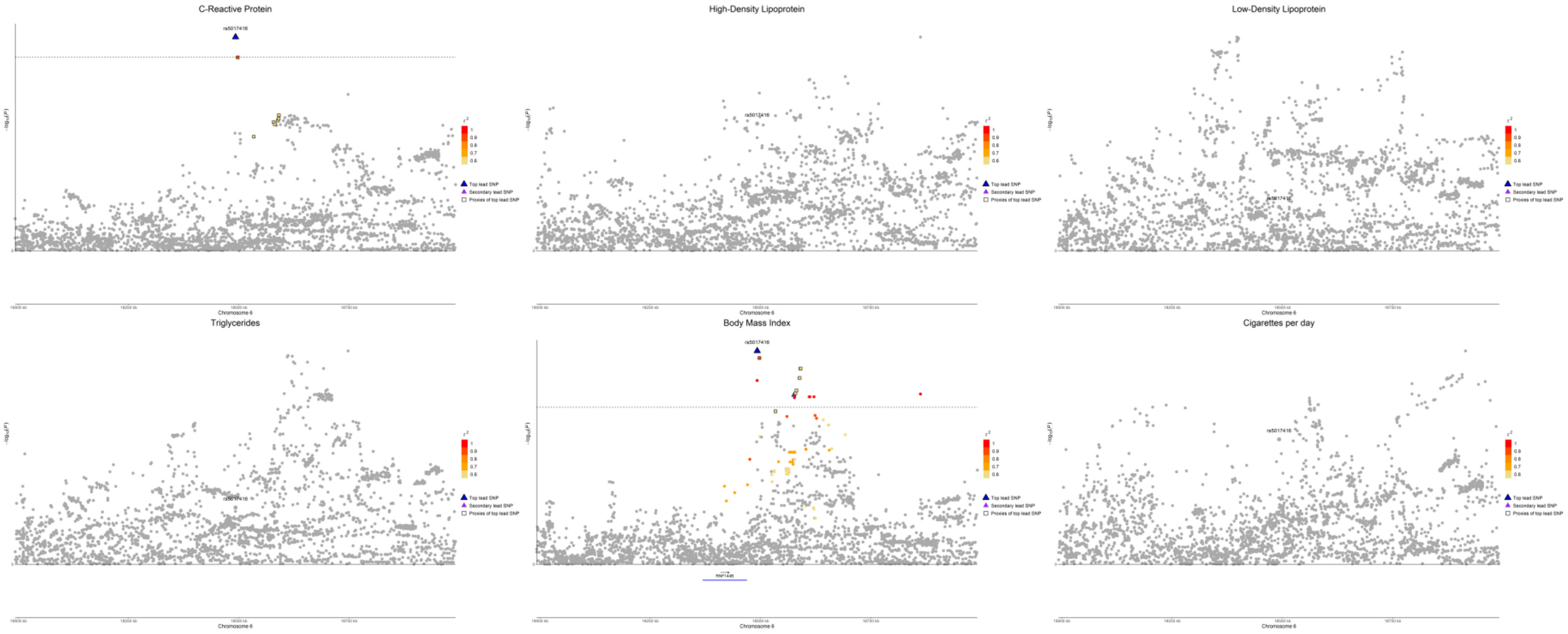

rs1490384

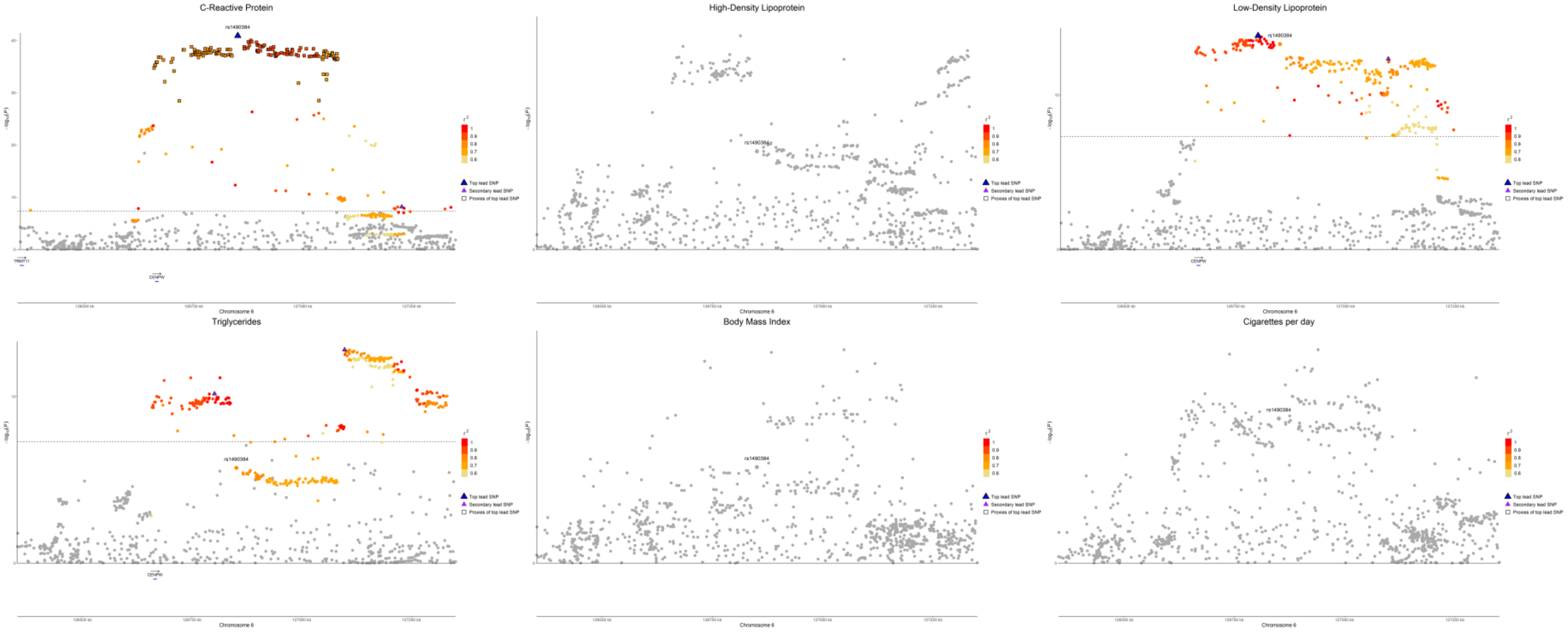

rs35237252

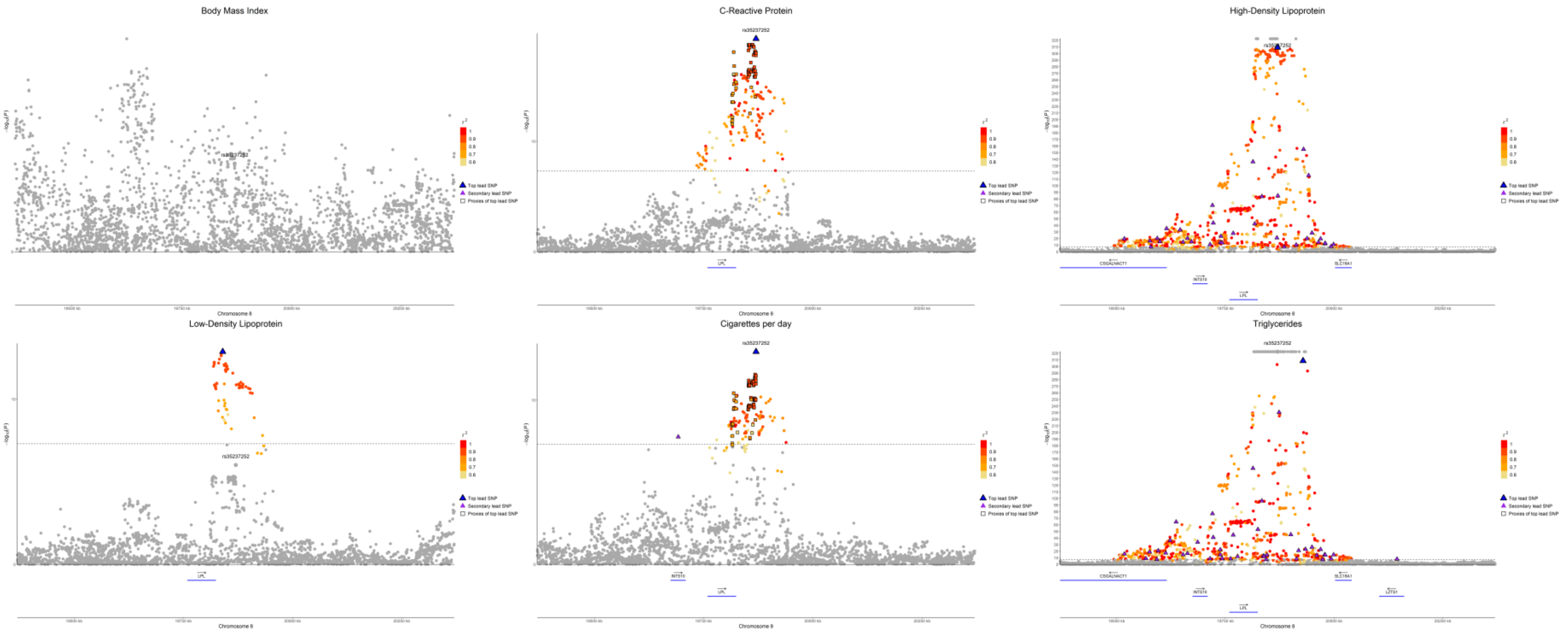

rs112875651

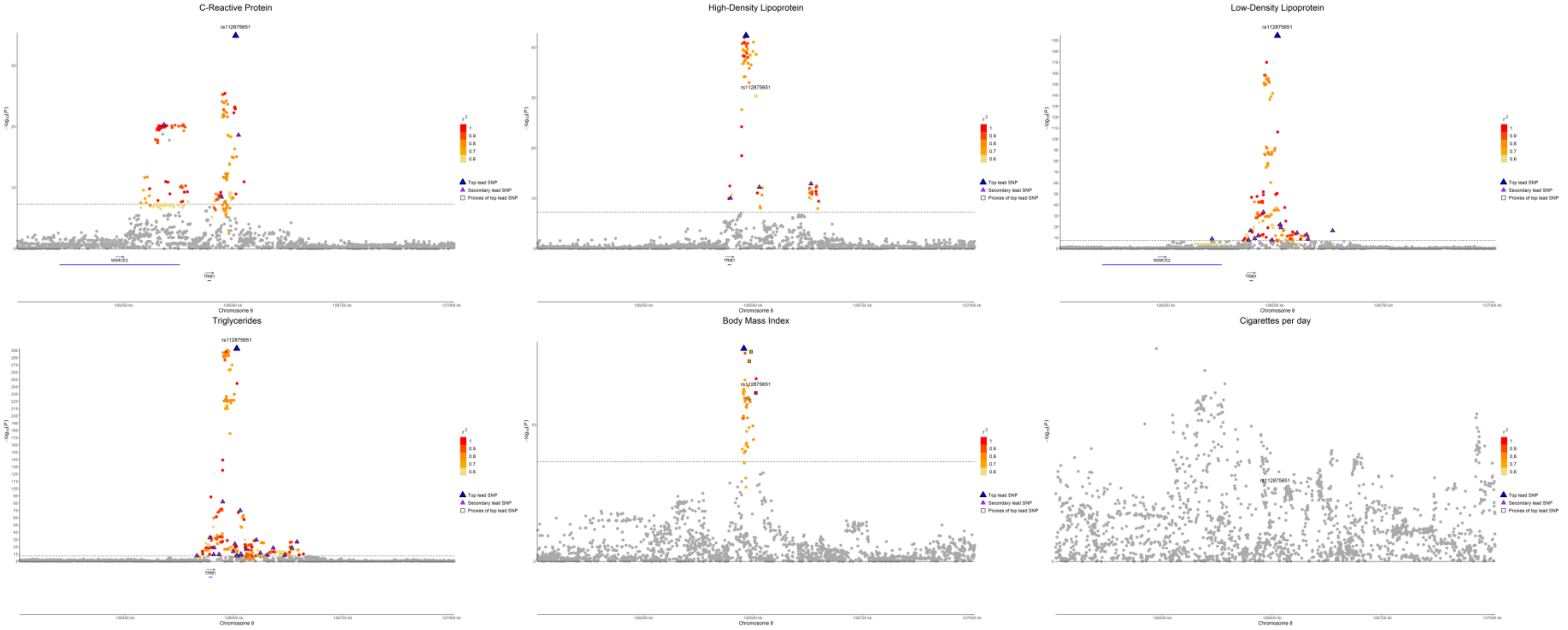

rs7031064

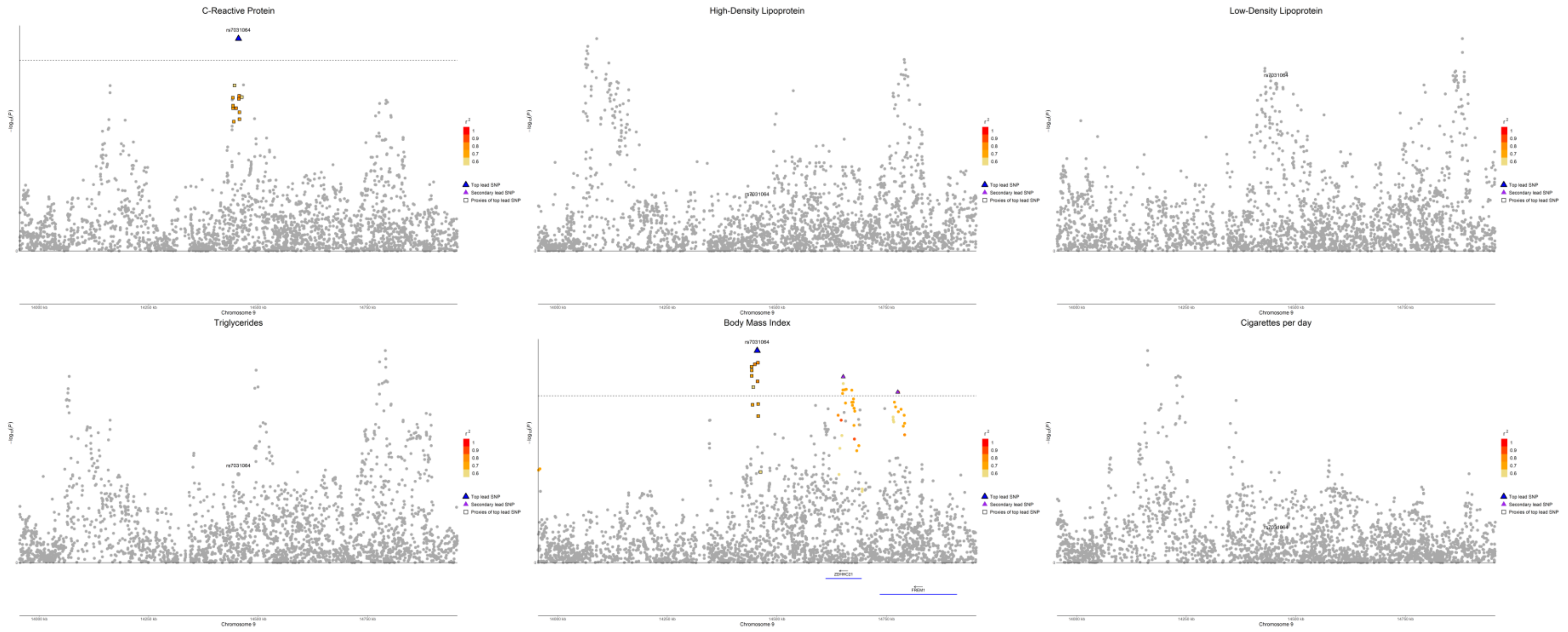

rs10968576

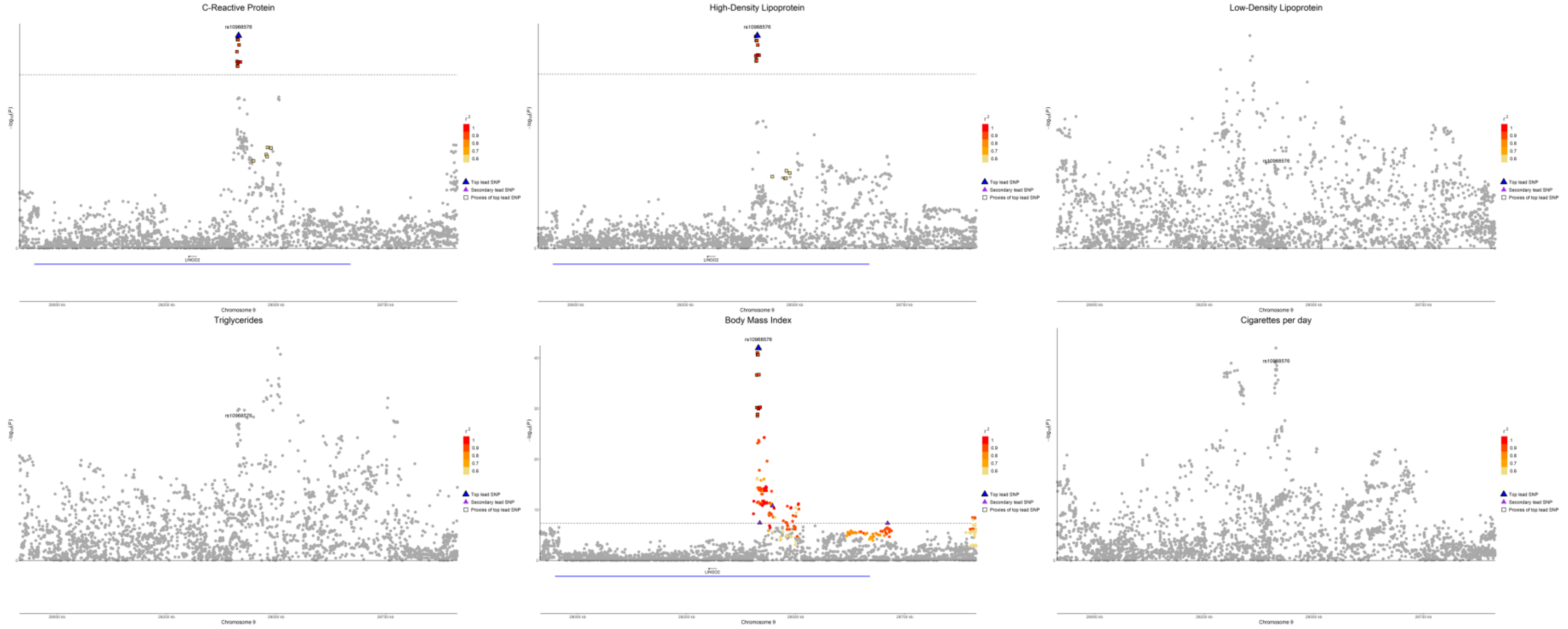

rs11012732

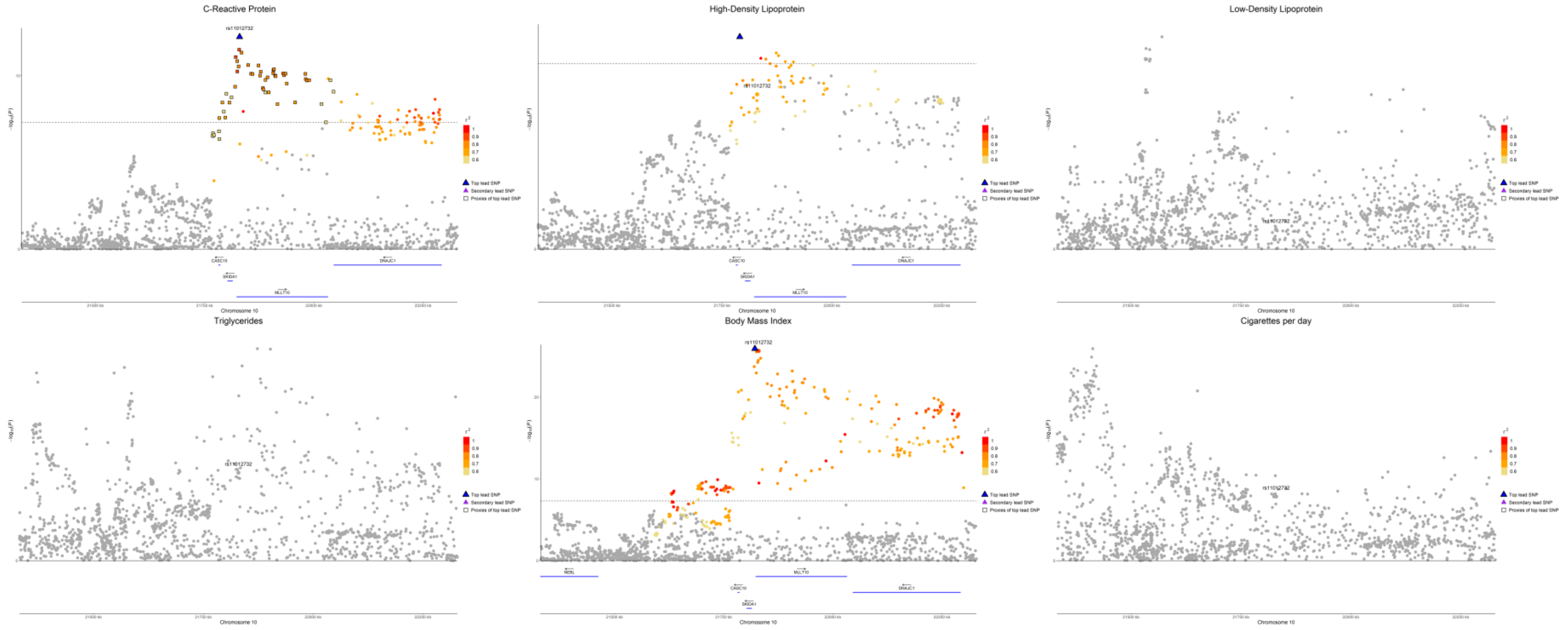

rs6486122

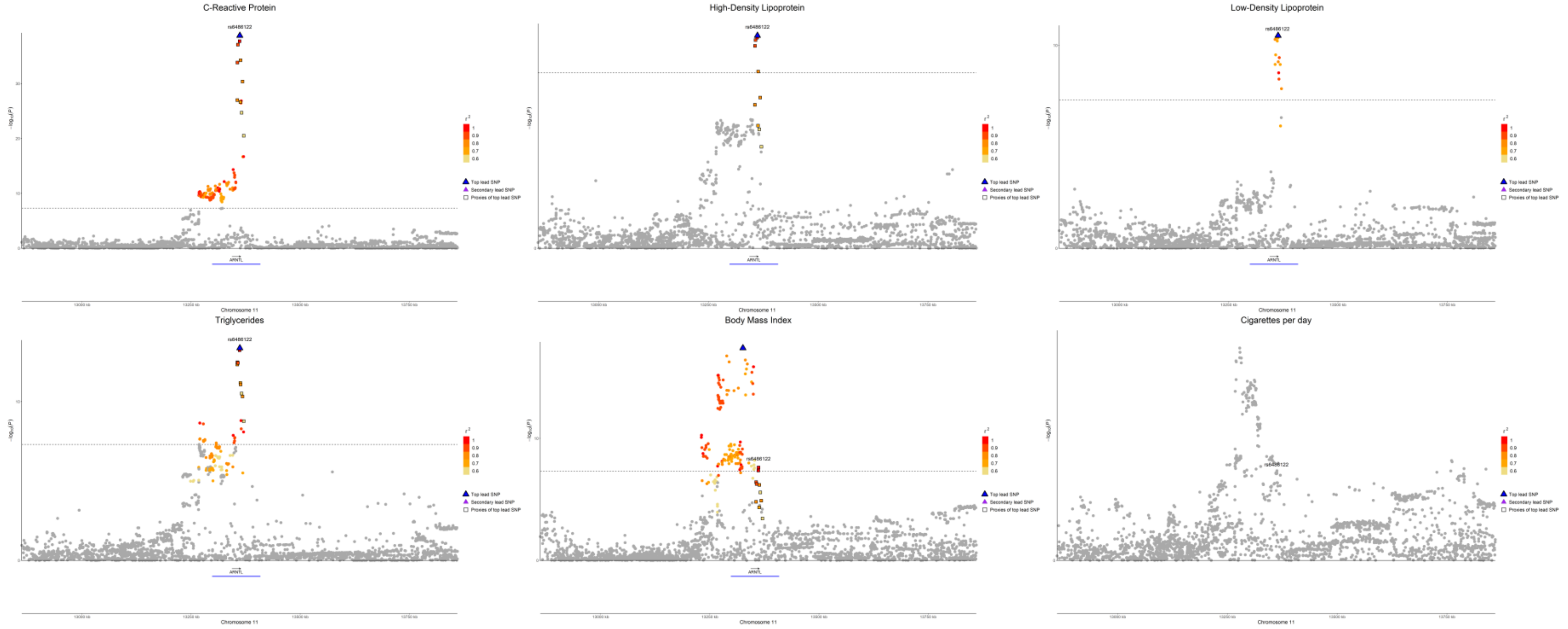

rs6265

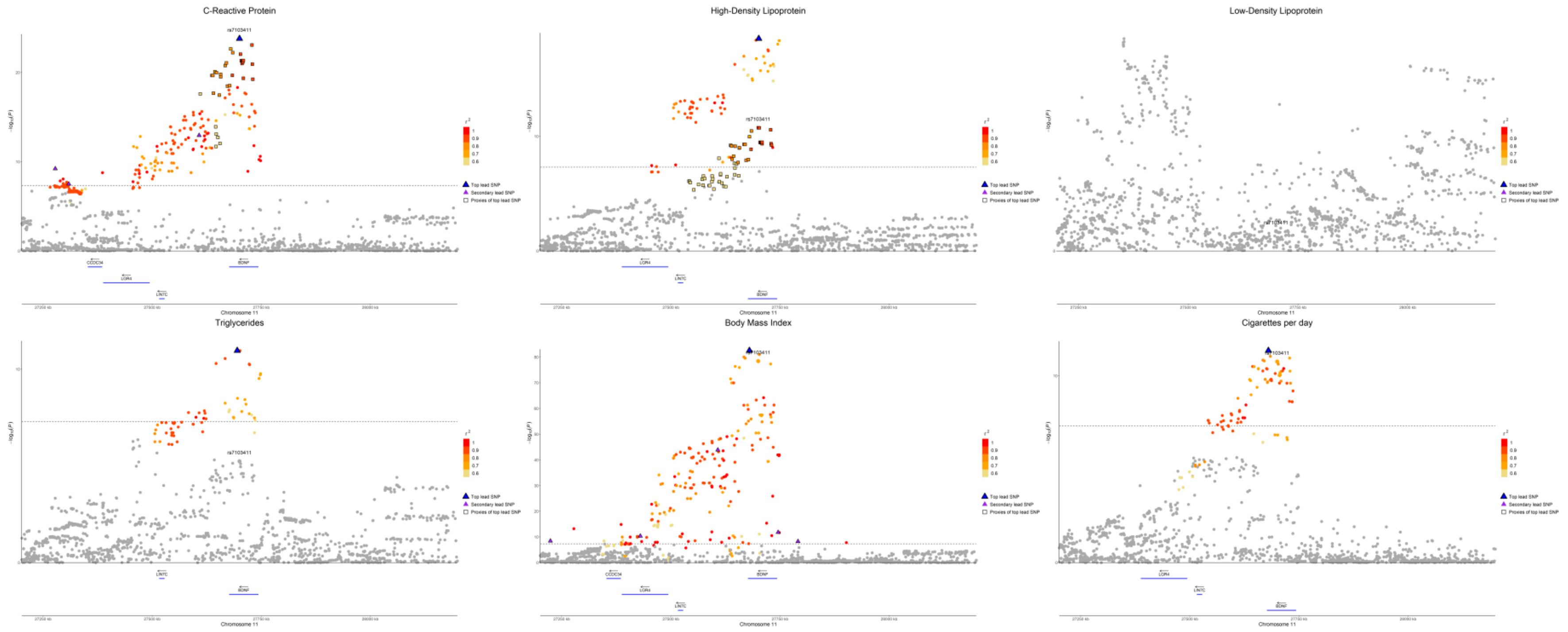

rs4755720

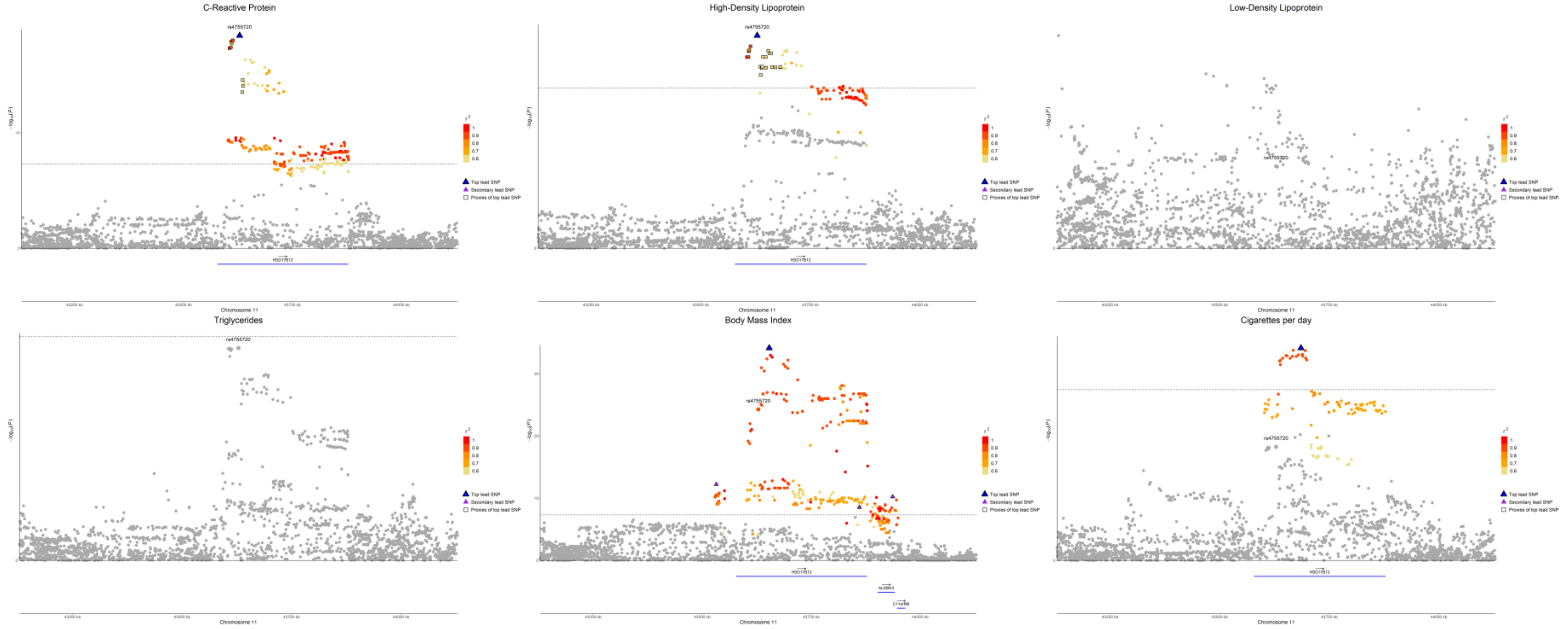

rs3741298

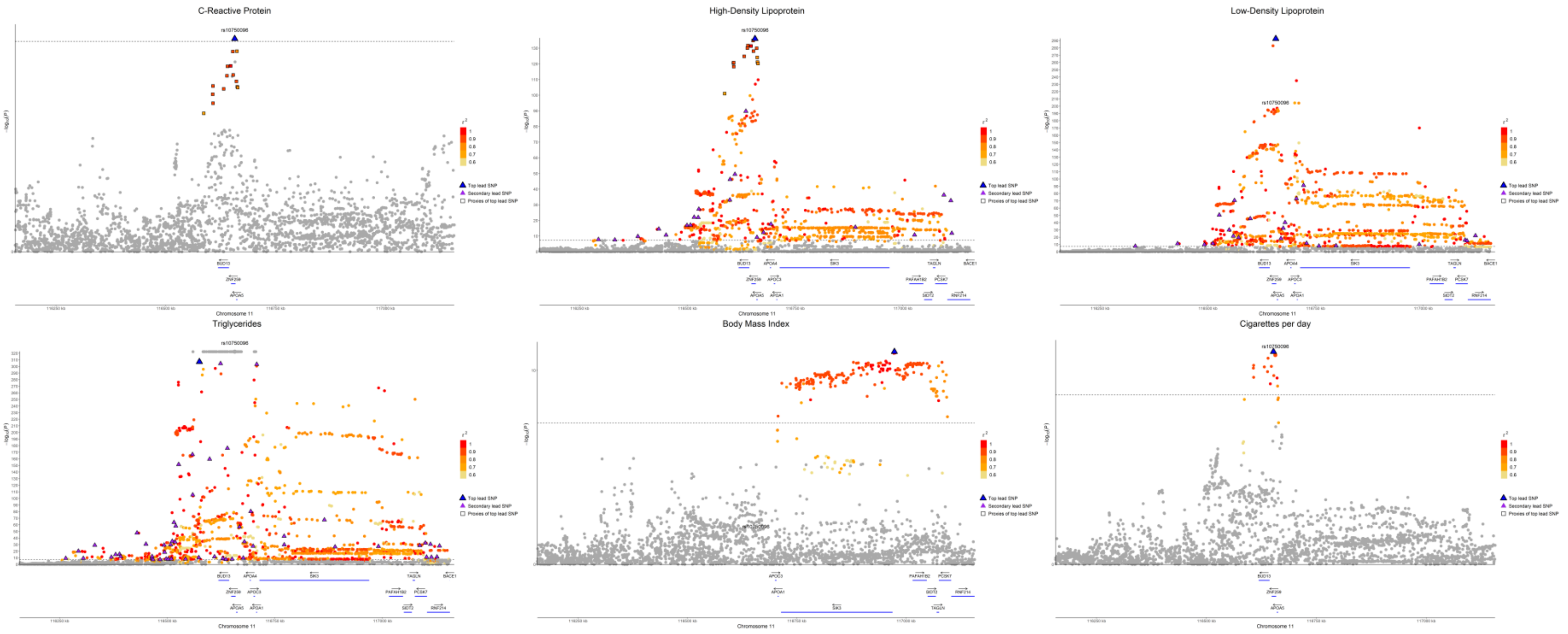

rs7138803

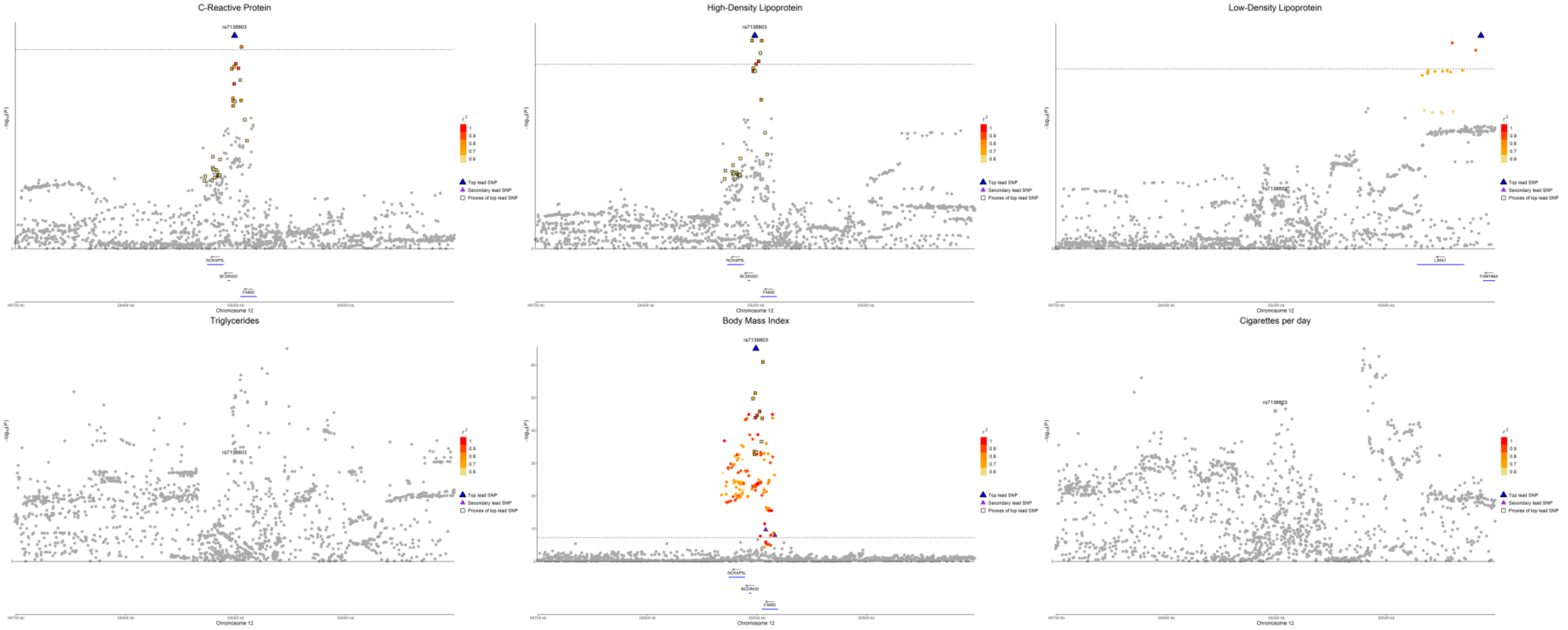

rs9604045

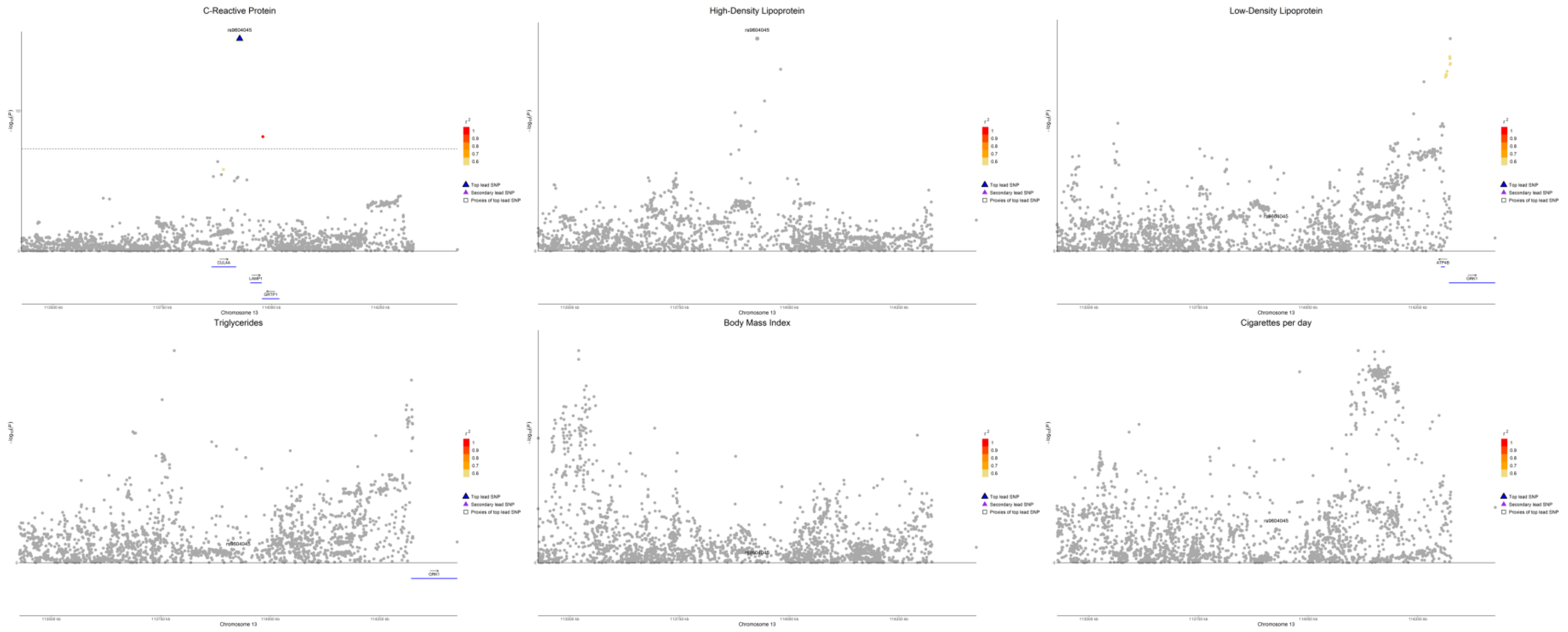

rs2239222

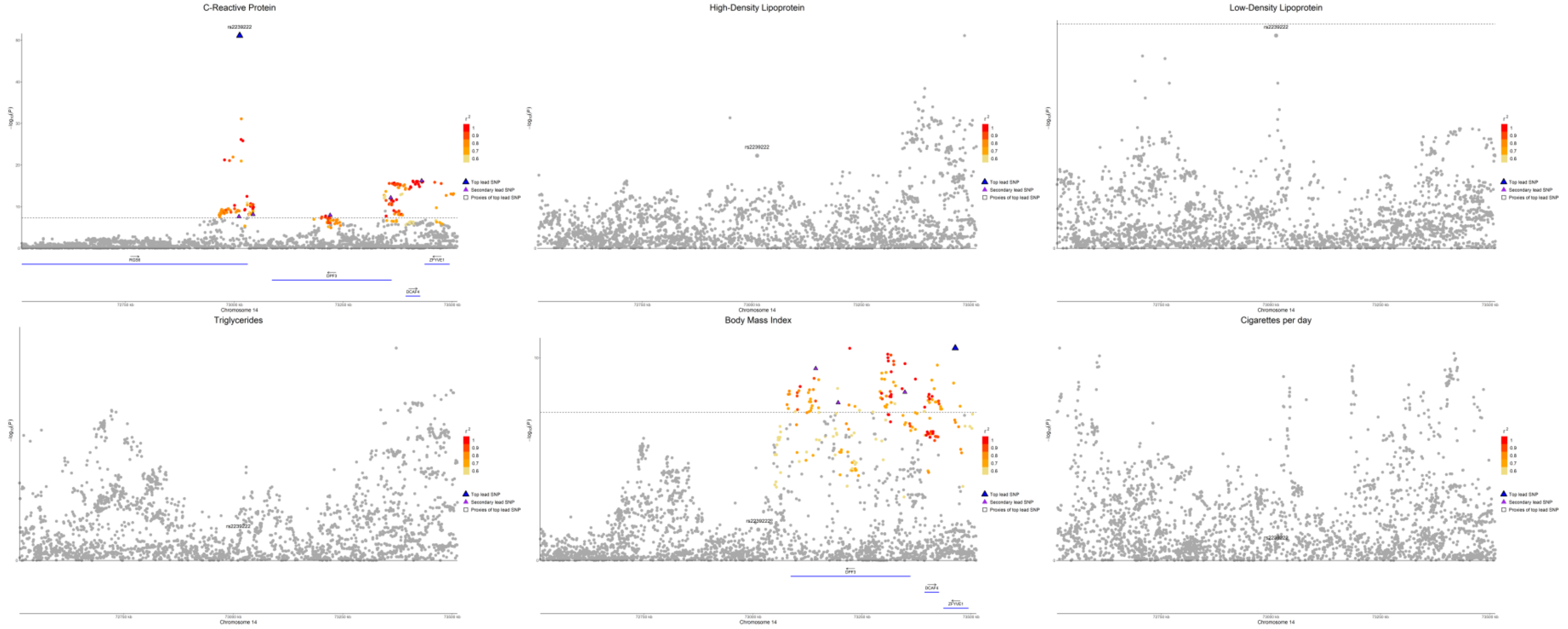

rs11635675

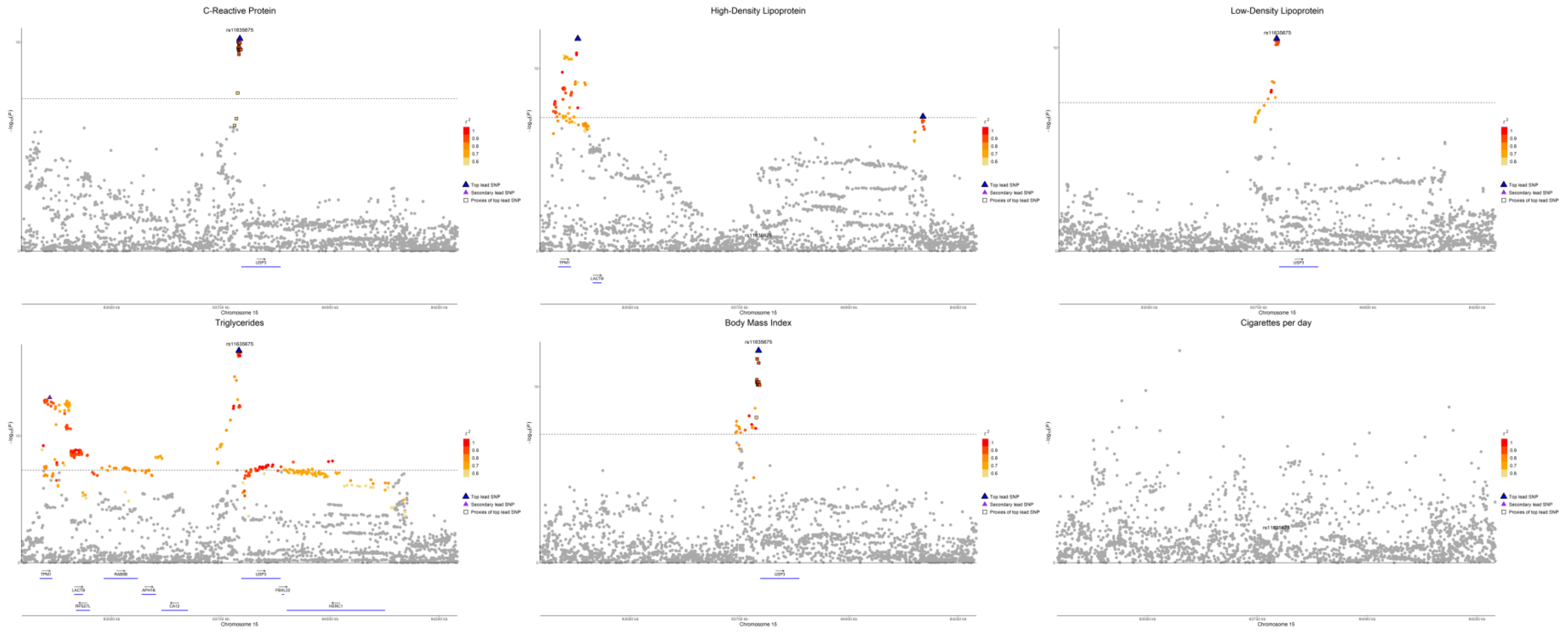

rs11852372

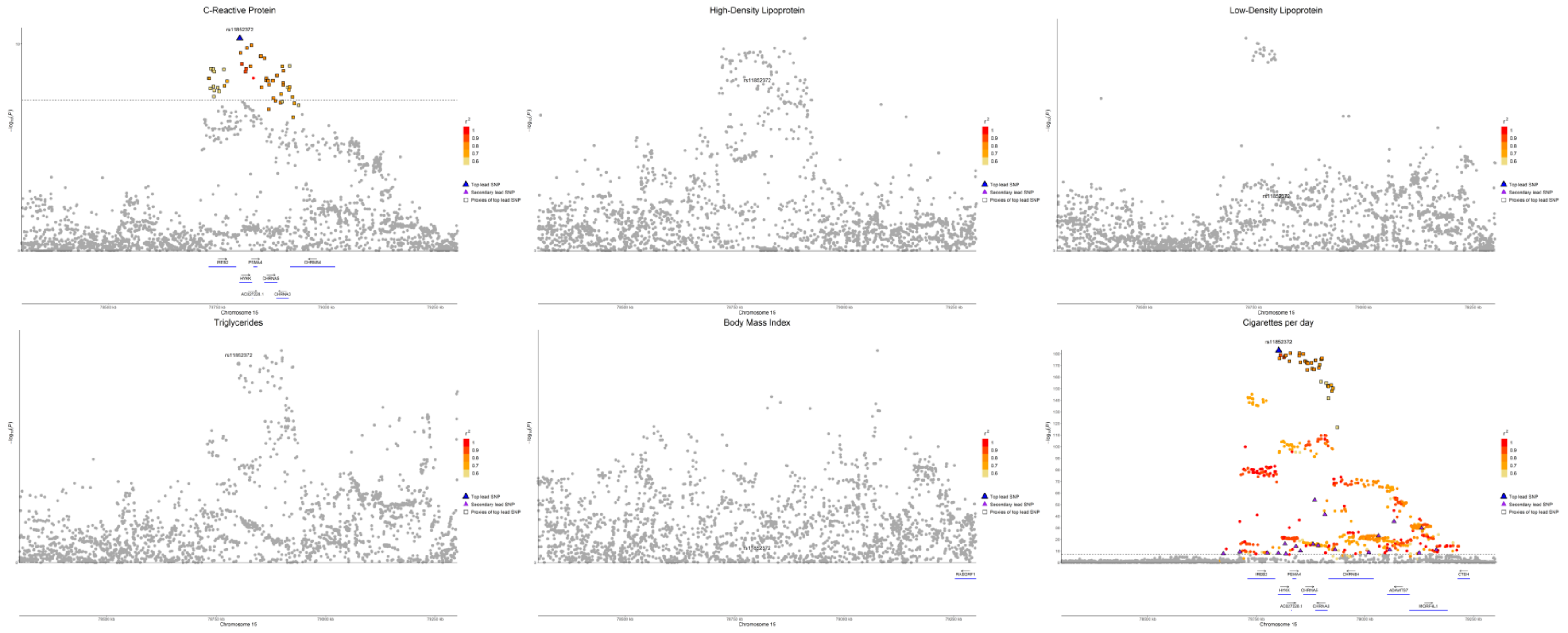

rs879620

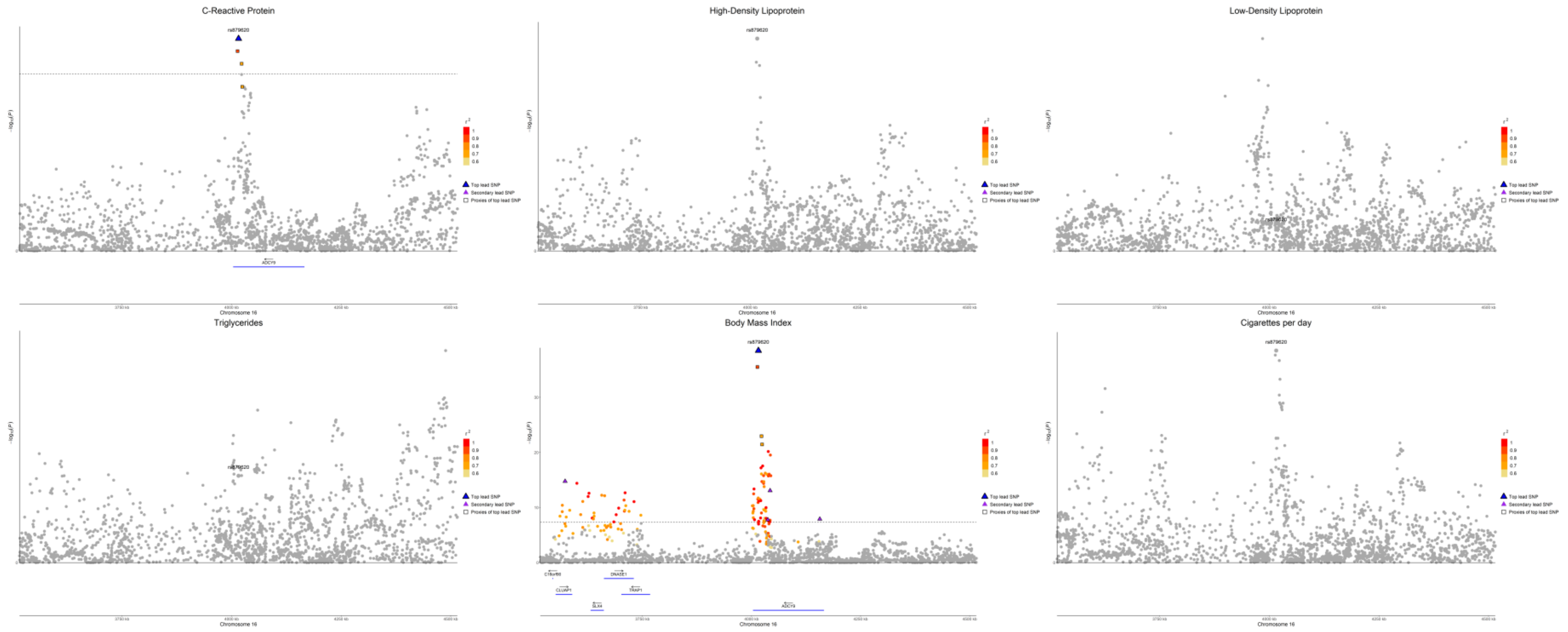

rs3814883

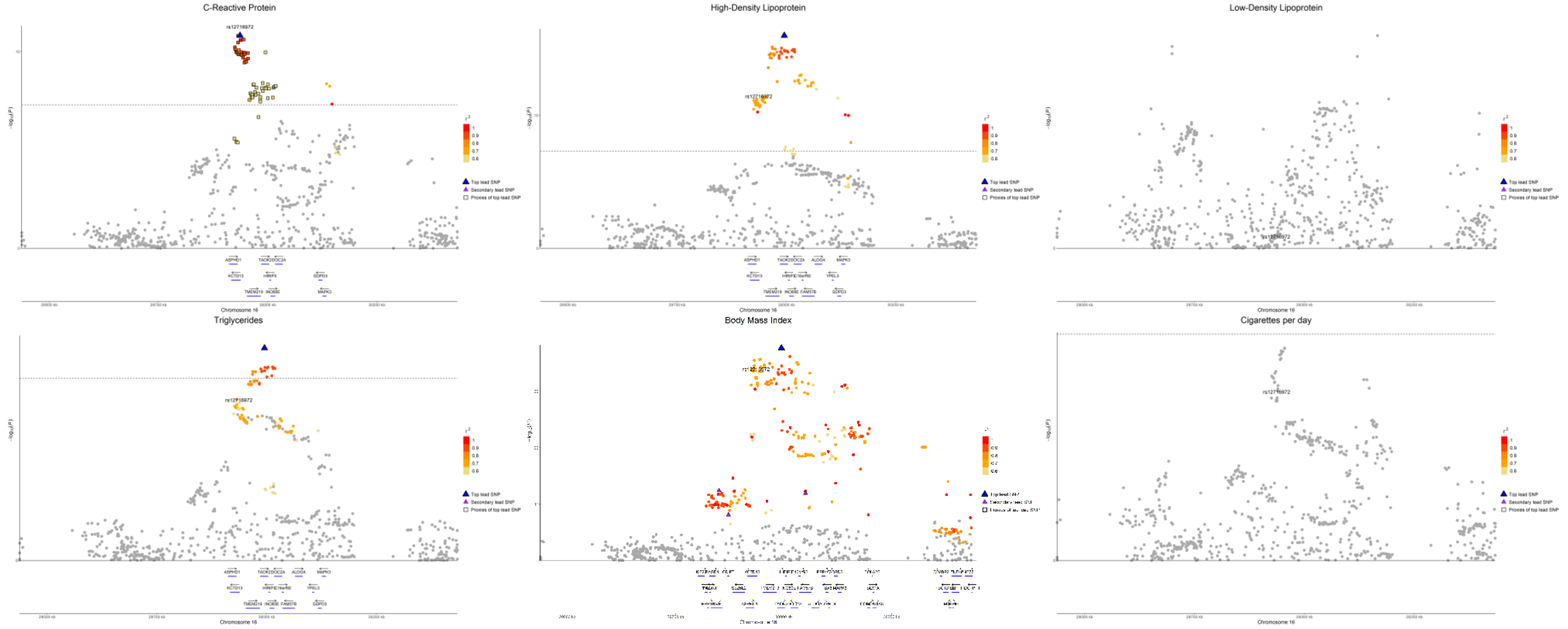

rs1421085

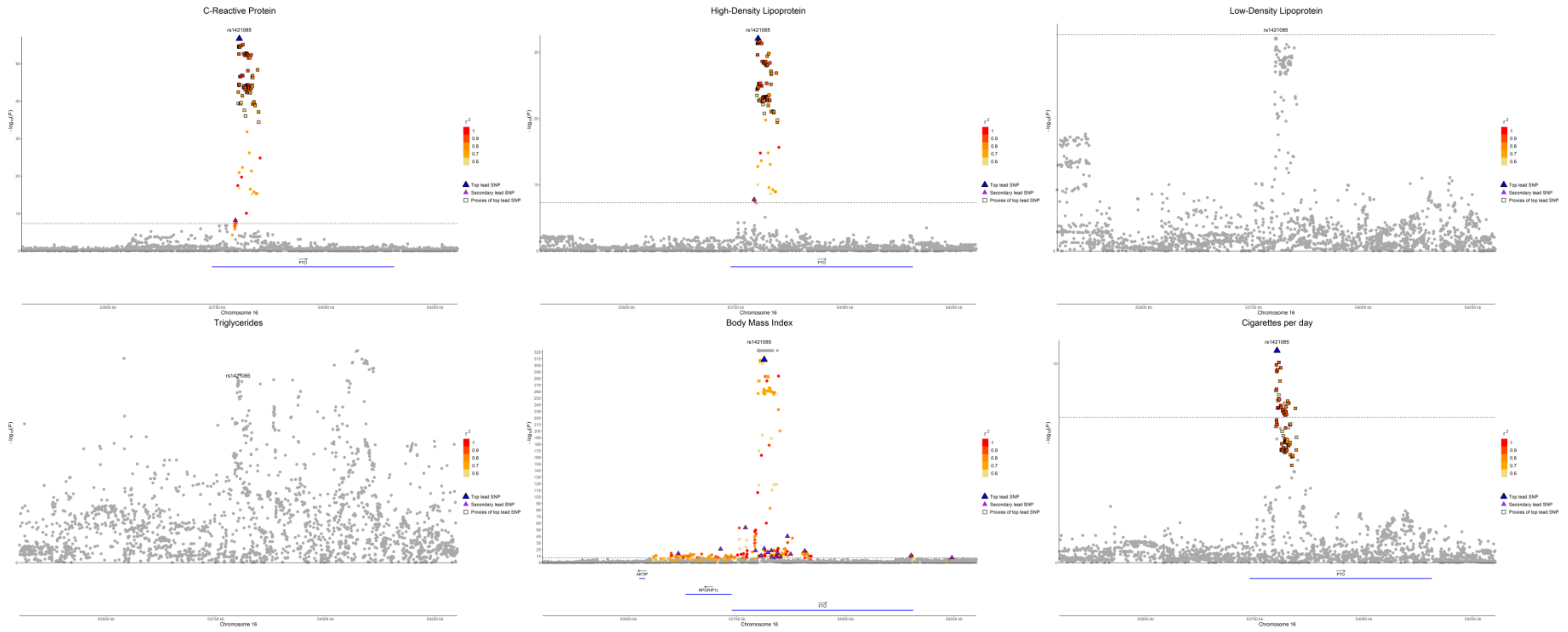

rs183130

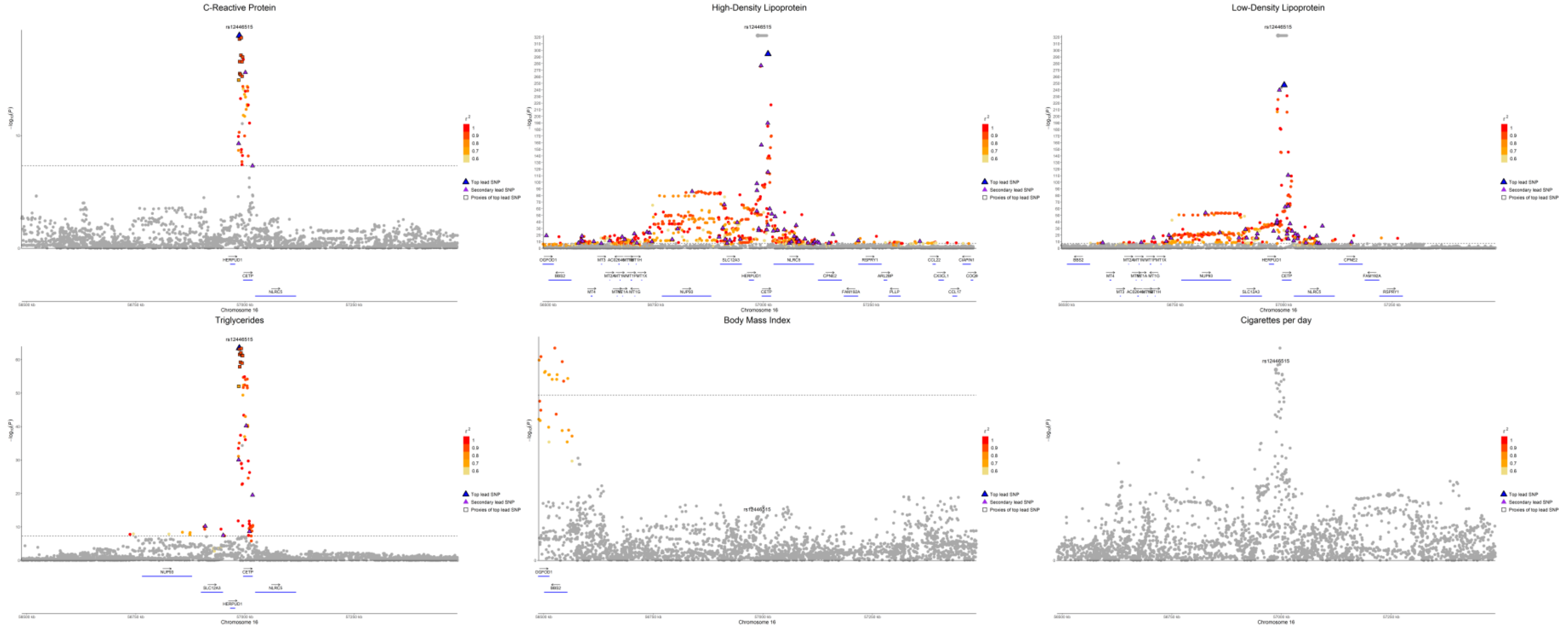

rs2000999

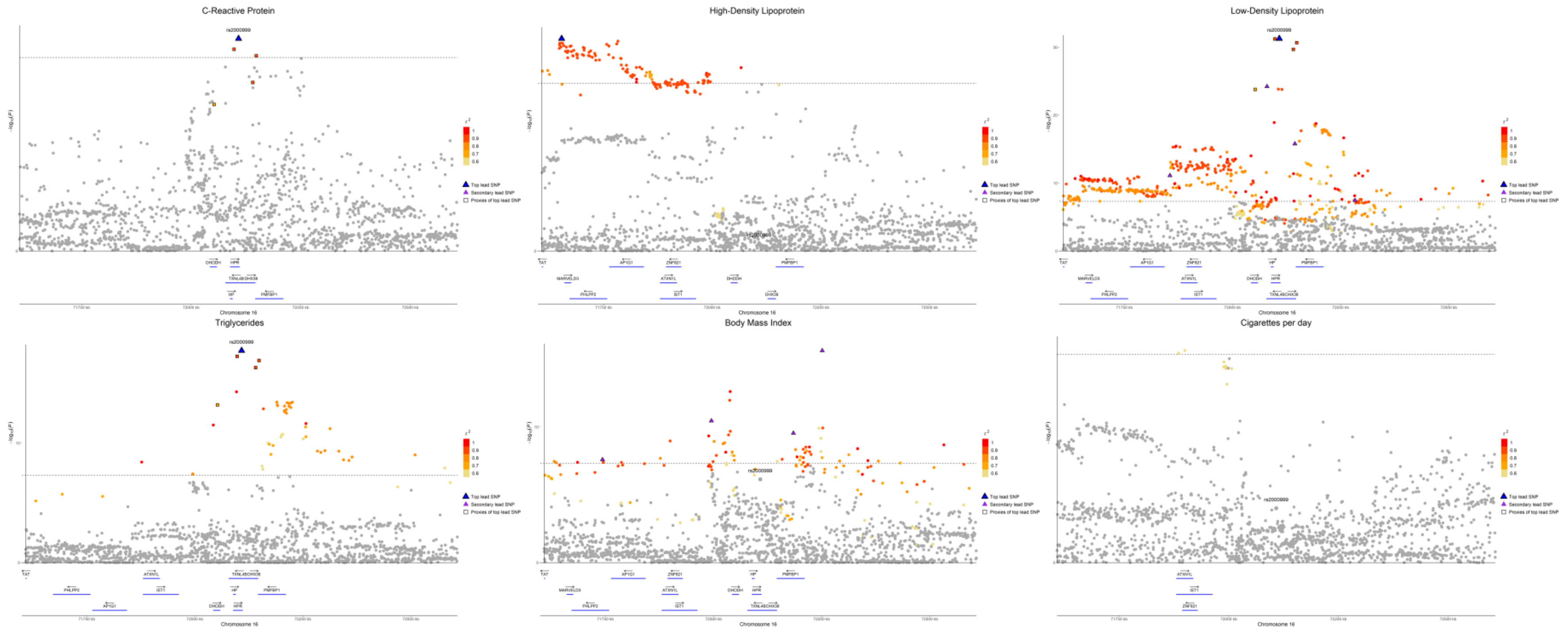

rs2925979

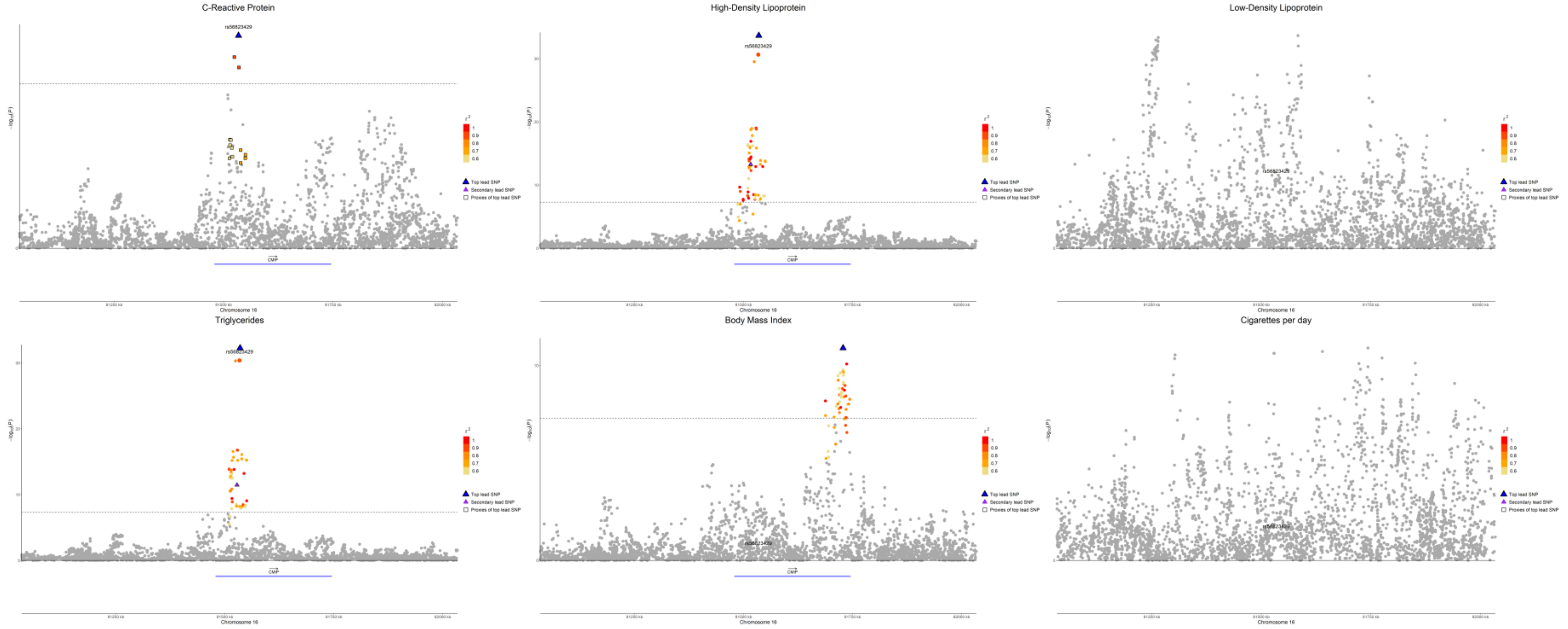

rs56113850

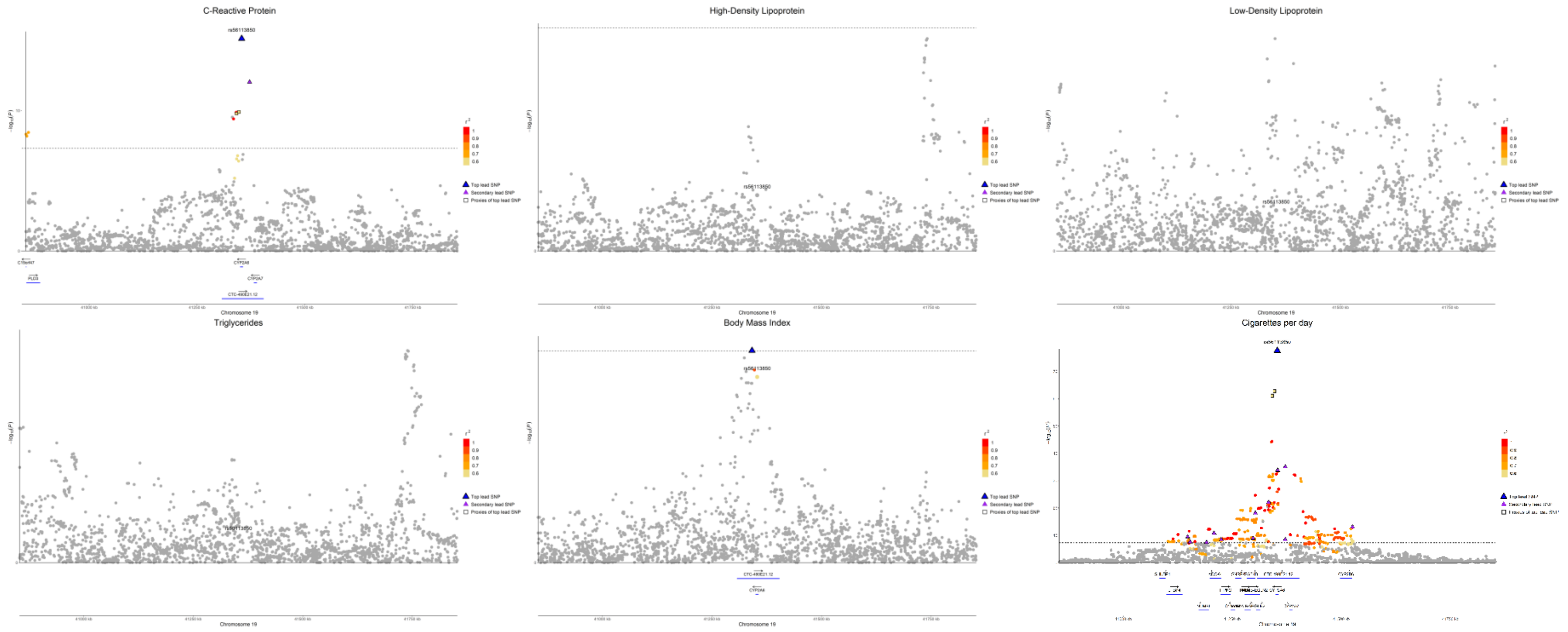

rs429358

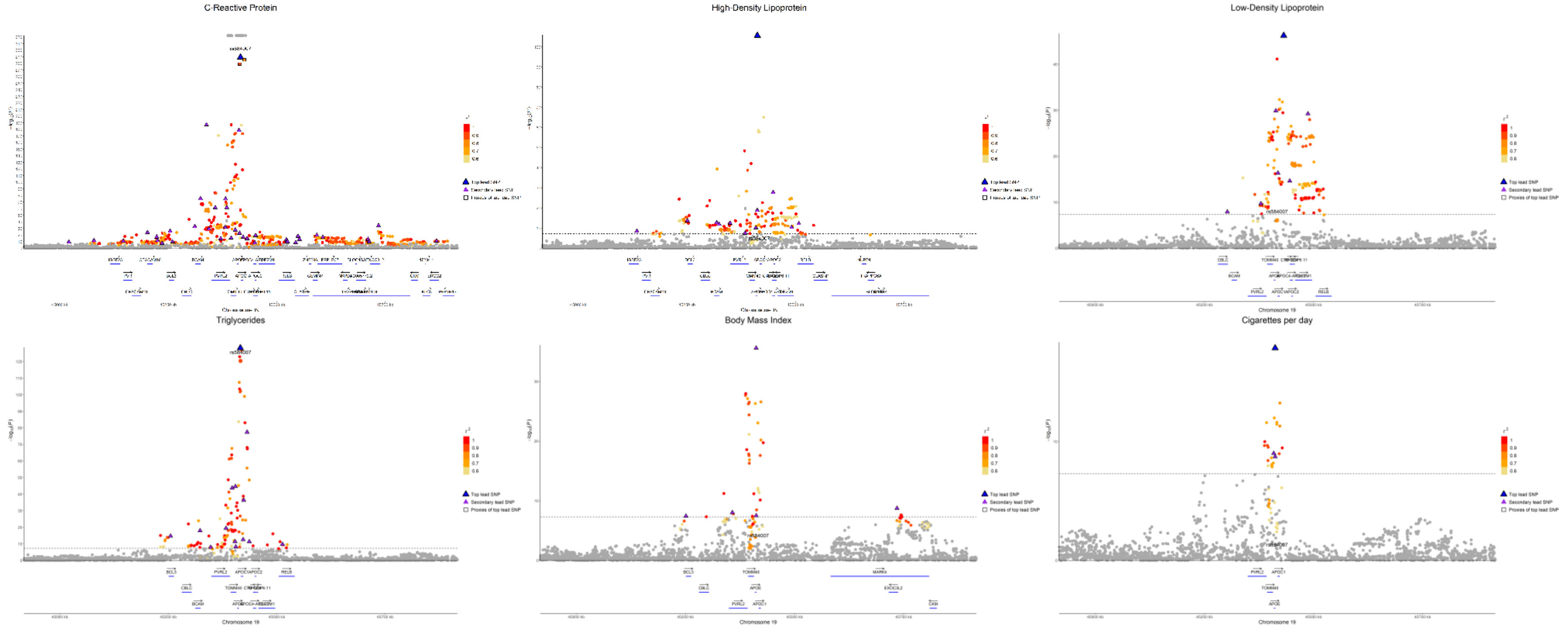

rs117113213

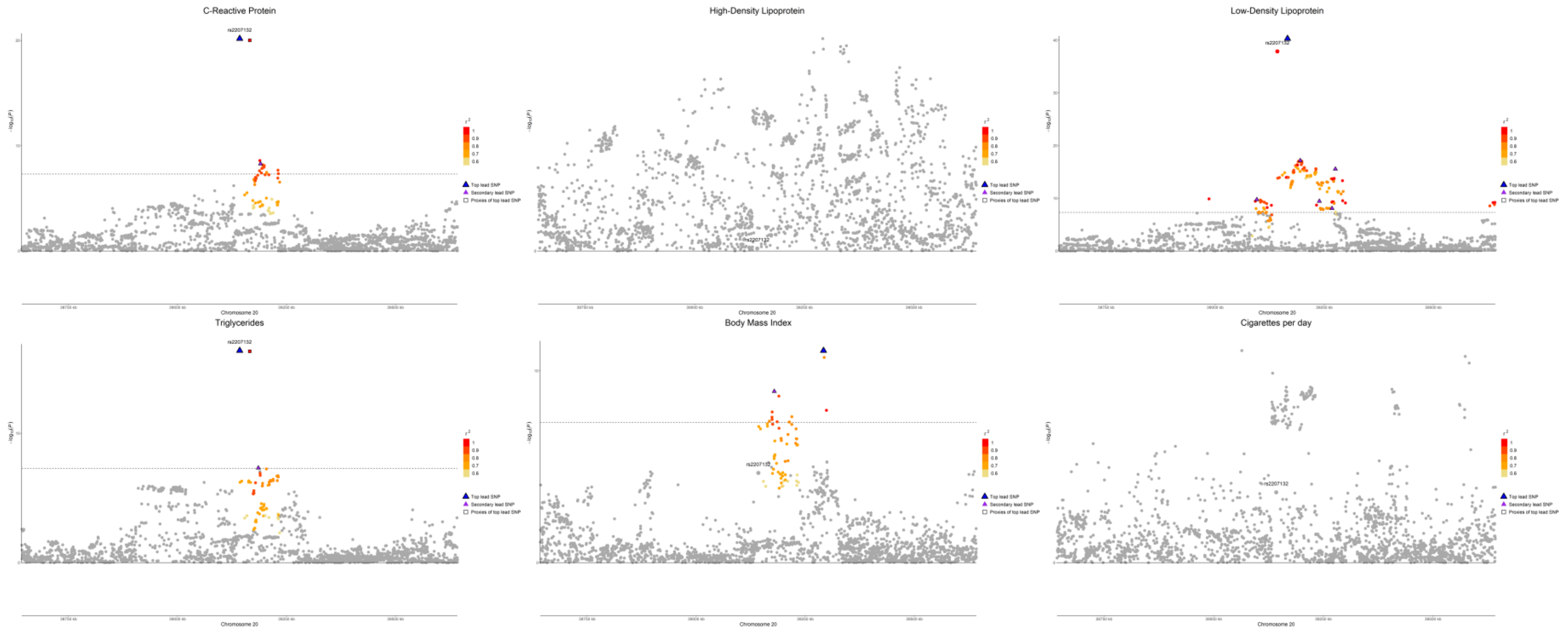

rs1800961

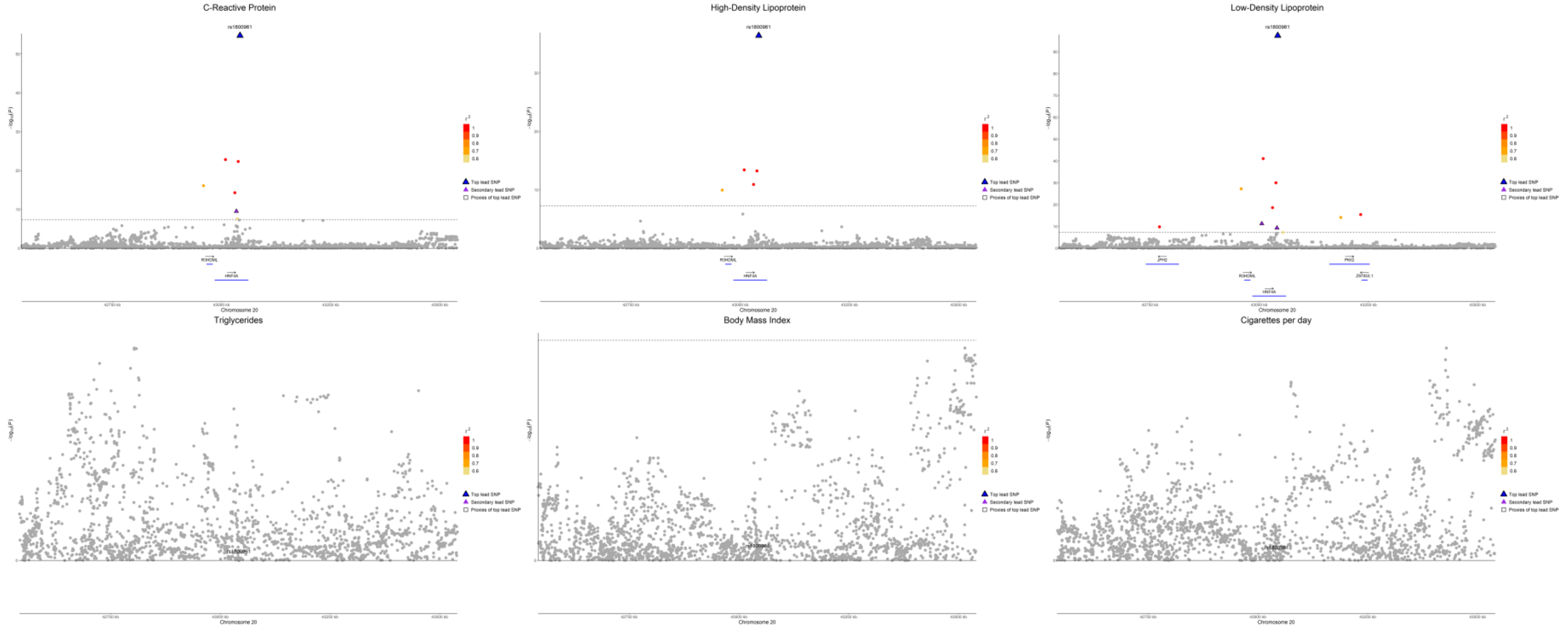

rs397092

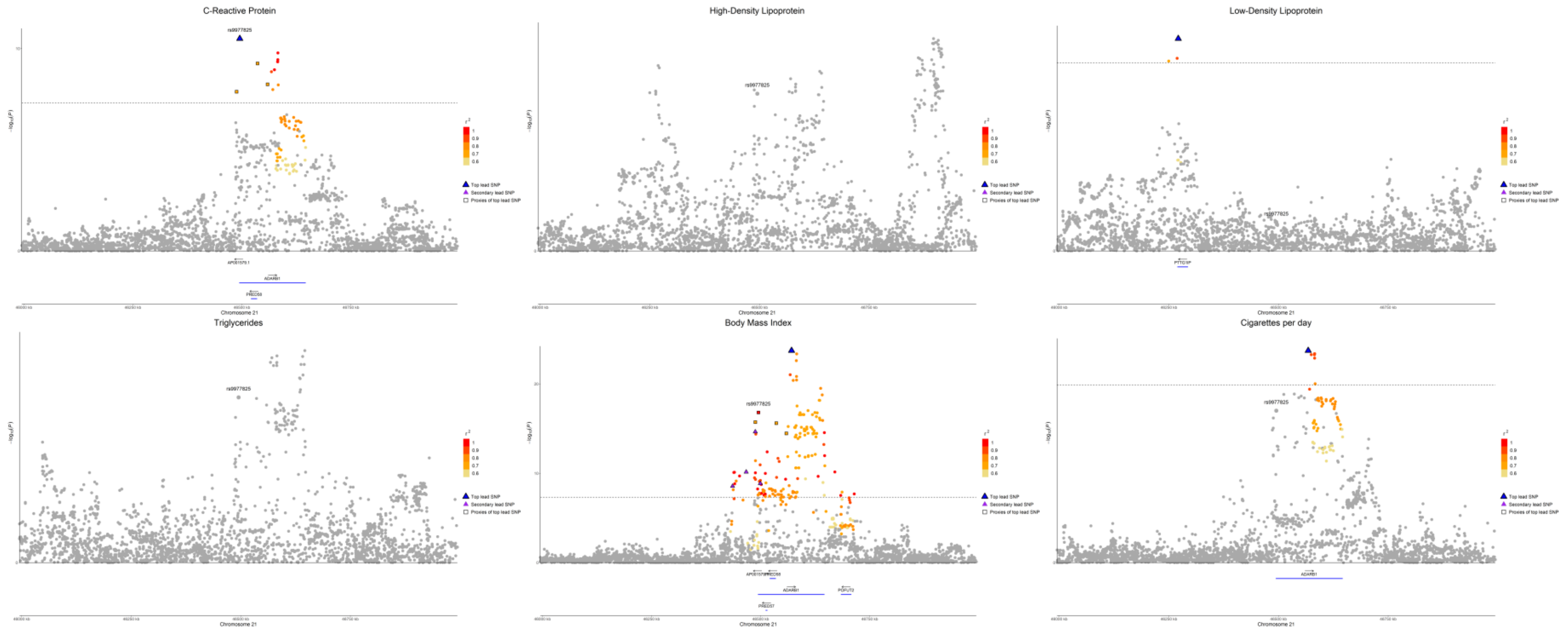

Supplementary Figure 7 *Regional plots of the 41 loci found to colocalize between C-Reactive Protein levels (CRP) and any of the other examined traits: High-Density Lipoprotein (HDL), Low-Density Lipoprotein (LDL), Triglycerides (TG), Body Mass Index (BMI), and Cigarettes per day (CPD). The P-values come from two-sided statistical tests.*
