# Supplementary material for: Pleiotropic genetic architecture and novel loci for C-reactive protein levels
Source: Nat Commun. 2022 Nov 14;13:6939. doi: 10.1038/s41467-022-34688-6 (PMC9663411; doi:10.1038/s41467-022-34688-6)
Supplement: Supplementary file 3 — Description of Additional Supplementary Files [file 41467_2022_34688_MOESM3_ESM.pdf]

Description of Additional Supplementary Files

File Name: Supplementary Data 1
Description: Genomic loci (top signals) associated with C-reactive protein in genome-wide significance level (P < 5e-8) indicated by multi-trait MTAG

File Name: Supplementary Data 2
Description: Secondary independent signals associated with C-reactive protein in genome-wide significance level (P < 5e-8) indicated by multi-trait MTAG

File Name: Supplementary Data 3
Description: Trait-specific independent (top + secondary) signals (P < 5e-8) indicated by multi-trait MTAG

File Name: Supplementary Data 4
Description: MTAG summary statistics of C-reactive protein independent SNPs (P < 5e-8) across all 6 examined traits

File Name: Supplementary Data 5
Description: C-reactive protein 41 additional independent signals (P < 5e-8) indicated by the bivariate MTAG analyses

File Name: Supplementary Data 6
Description: Genes associated with C-reactive protein levels (CRP; N=1816), high density lipoprotein levels (HDL; N=1451), low density lipoprotein levels (LDL; N=984), triglyceride levels (TG; N=1178), body mass index (BMI; N=2949) and cigarettes per day (CPD; N=290)

File Name: Supplementary Data 7
Description: Genes associated with C-reactive protein levels (CRP) and any of the other examined traits: high density lipoprotein levels (HDL), low density lipoprotein levels (LDL), triglyceride levels (TG), body mass index (BMI) and cigarettes per day (CPD)

File Name: Supplementary Data 8
Description: Gene-sets associated with C-reactive protein (CRP; N=19); high density lipoprotein levels (HDL; N=51); low density lipoprotein levels (LDL; N=79); triglyceride levels (TG; N=25); body mass index (BMI; N=25) and cigarettes per day (CPD; N=16) in a Bonferrroni significance level

File Name: Supplementary Data 9
Description: Gene-property analysis results for C-reactive protein (CRP), high density lipoprotein levels (HDL), low density lipoprotein levels (LDL), triglyceride levels (TG), body mass index (BMI) and cigarettes per day (CPD) using 30 general tissue types from GTEx v8

File Name: Supplementary Data 10
Description: Gene-property analysis results for C-reactive protein (CRP), high density lipoprotein levels (HDL), low density lipoprotein levels (LDL), triglyceride levels (TG), body

mass index (BMI) and cigarettes per day (CPD) using 54 more specific tissue types from GTEx v8

File Name: Supplementary Data 11
Description: C-reactive protein (CRP) associated loci which colocalize (PP > 0.5) between CRP and any combination of the other examined traits: high density lipoproteins (HDL), low density lipoproteins (LDL), Triglycerides (TG), body mass index (BMI) and cigarettes per day (CPD)

File Name: Supplementary Data 12
Description: C-reactive protein (CRP) associated SNPs with discordant direction of effect between CRP and any combination of the other examined traits: high density lipoprotein levels (HDL), low density lipoproteins (LDL), triglyceride levels (TG), body mass index (BMI) and cigarettes per day (CPD)

File Name: Supplementary Data 13
Description: PheWAS results for the 12 colocalized SNPs with discordant genetic effect between C-reactive protein levels (CRP) and any combination of the other examined traits: high density lipoprotein levels (HDL), low density lipoproteins (LDL), triglyceride levels (TG), body mass index (BMI) and cigarettes per day (CPD)

File Name: Supplementary Data 14
Description: Trait-specific results from Mendelian Randomization analysis using C-reactive protein levels (CRP), high density lipoprotein levels (HDL), low density lipoprotein levels (LDL), triglyceride levels (TG), body mass index (BMI) or cigarettes per day (CPD) as exposure

File Name: Supplementary Data 15
Description: Information of the included studies used in MTAG analyses

File Name: Supplementary Data 16
Description: Information of the studies used in Mendelian Randomization analysis as outcomes
