# Supplementary material for: Pleiotropic genetic architecture and novel loci for C-reactive protein levels
Source: Nat Commun. 2022 Nov 14;13:6939. doi: 10.1038/s41467-022-34688-6 (PMC9663411; doi:10.1038/s41467-022-34688-6)
Supplement: Supplementary file 5 — Reporting Summary [file 41467_2022_34688_MOESM5_ESM.pdf]

nature portfolio

Corresponding author(s): Fotios Koskeridis

Last updated by author(s): Oct 20, 2022

Reporting Summary

Nature Portfolio wishes to improve the reproducibility of the work that we publish. This form provides structure for consistency and transparency in reporting. For further information on Nature Portfolio policies, see our Editorial Policies and the Editorial Policy Checklist.

Statistics

For all statistical analyses, confirm that the following items are present in the figure legend, table legend, main text, or Methods section.

n/a	Confirmed	
☐	☒	The exact sample size (*n*) for each experimental group/condition, given as a discrete number and unit of measurement
☐	☒	A statement on whether measurements were taken from distinct samples or whether the same sample was measured repeatedly
☐	☒	The statistical test(s) used AND whether they are one- or two-sided<br>*Only common tests should be described solely by name; describe more complex techniques in the Methods section.*
☐	☒	A description of all covariates tested
☐	☒	A description of any assumptions or corrections, such as tests of normality and adjustment for multiple comparisons
☐	☒	A full description of the statistical parameters including central tendency (e.g. means) or other basic estimates (e.g. regression coefficient) AND variation (e.g. standard deviation) or associated estimates of uncertainty (e.g. confidence intervals)
☐	☒	For null hypothesis testing, the test statistic (e.g. *F*, *t*, *r*) with confidence intervals, effect sizes, degrees of freedom and *P* value noted<br>*Give P values as exact values whenever suitable.*
☐	☒	For Bayesian analysis, information on the choice of priors and Markov chain Monte Carlo settings
☒	☐	For hierarchical and complex designs, identification of the appropriate level for tests and full reporting of outcomes
☐	☒	Estimates of effect sizes (e.g. Cohen's *d*, Pearson's *r*), indicating how they were calculated

Our web collection on statistics for biologists contains articles on many of the points above.

Software and code

Policy information about availability of computer code

Data collection	No software or code was used for the data collection in this study
Data analysis	MTAG, R v4.1.0, FUMA v1.3.6a, PLINK v1.9, ANNOVAR (2017), MAGMA v1.08, HyPrColoc v1.0.0, PheWAS v0.99.5-5, TwoSampleMR v0.4.26

For manuscripts utilizing custom algorithms or software that are central to the research but not yet described in published literature, software must be made available to editors and reviewers. We strongly encourage code deposition in a community repository (e.g. GitHub). See the Nature Portfolio guidelines for submitting code & software for further information.

Data

Policy information about availability of data

All manuscripts must include a data availability statement. This statement should provide the following information, where applicable:

- Accession codes, unique identifiers, or web links for publicly available datasets
- A description of any restrictions on data availability
- For clinical datasets or third party data, please ensure that the statement adheres to our policy

The summary statistics of all GWAS used in this study are publicly available from GWAS Catalog (CRP: https://www.ebi.ac.uk/gwas/studies/GCST90029070; BMI: https://www.ebi.ac.uk/gwas/studies/GCST009004; CPD: https://www.ebi.ac.uk/gwas/studies/GCST007459) or Neale lab (lipids: http://www.nealelab.is/uk-biobank). The MTAG summary statistics generated in this study have been deposited in NHGRI-EBI GWAS Catalog under accession codes GCST90179146 (CRP; https://www.ebi.ac.uk/gwas/studies/GCST90179146), GCST90179147 (HDL; https://www.ebi.ac.uk/gwas/studies/GCST90179147), GCST90179148 (LDL; https://

www.ebi.ac.uk/gwas/studies/GCST90179148), GCST90179149 (TG; https://www.ebi.ac.uk/gwas/studies/GCST90179149), GCST90179150 (BMI; https://www.ebi.ac.uk/gwas/studies/GCST90179150) and GCST90179151 (CPD; https://www.ebi.ac.uk/gwas/studies/GCST90179151). All other data generated in this study are provided with this published article (and its supplementary information files).

Human research participants

Policy information about studies involving human research participants and Sex and Gender in Research.

Reporting on sex and gender	This study used summary statistics from previously published GWAS studies including males and females. Any information regarding the sex and gender definition is described in the respective study. References to the included studies are provided in Supplementary Table 15. No sex- or gender-based analysis was performed in this study.
Population characteristics	This study used summary statistics from previously published GWAS studies on population of European ancestry. The population characteristics of the included studies are described in the respective study and references to the included studies are provided in Supplementary Table 15
Recruitment	This study used summary statistics from previously published GWAS studies. No additional recruitment was performed for the purposes of those analyses.
Ethics oversight	This study used summary statistics from previously published GWAS studies. The ethical approvals for each included GWAS study are described in the respective study and references to the included studies are provided in Supplementary Table 15

Note that full information on the approval of the study protocol must also be provided in the manuscript.

Field-specific reporting

Please select the one below that is the best fit for your research. If you are not sure, read the appropriate sections before making your selection.

☒ Life sciences ☐ Behavioural & social sciences ☐ Ecological, evolutionary & environmental sciences

For a reference copy of the document with all sections, see nature.com/documents/nr-reporting-summary-flat.pdf

Life sciences study design

All studies must disclose on these points even when the disclosure is negative.

Sample size	This study used summary statistics from previously published GWAS studies. As a rule, the largest GWAS of European ancestry for the respective trait was used.
Data exclusions	All insertion and deletion polymorphisms, rare variants (MAF < 0.01), variants with a sample size less than 2/3 of the 90th percentile and palindromic SNPs were excluded from the analysis.
Replication	The genetic loci, found from multi-trait analyses to be associated with CRP and any of the other examined traits, were further investigated with colocalization analysis. Our manuscript focuses only on replicated loci.
Randomization	This study used summary statistics from previously published GWAS studies. Randomization is not applicable based on the design of our study
Blinding	This study used summary statistics from previously published GWAS studies. Blinding is not applicable based on the design of our study

Reporting for specific materials, systems and methods

We require information from authors about some types of materials, experimental systems and methods used in many studies. Here, indicate whether each material, system or method listed is relevant to your study. If you are not sure if a list item applies to your research, read the appropriate section before selecting a response.

Materials & experimental systems

n/a	Involved in the study
☒	☐ Antibodies
☒	☐ Eukaryotic cell lines
☒	☐ Palaeontology and archaeology
☒	☐ Animals and other organisms
☒	☐ Clinical data
☒	☐ Dual use research of concern

Methods

n/a	Involved in the study
☒	☐ ChIP-seq
☒	☐ Flow cytometry
☒	☐ MRI-based neuroimaging
